# A cross-species spatiotemporal proteomic analysis identifies UBE3A-dependent signaling pathways and targets

**DOI:** 10.1038/s41380-022-01484-z

**Published:** 2022-03-09

**Authors:** Nikhil J. Pandya, Sonja Meier, Stefka Tyanova, Marco Terrigno, Congwei Wang, A. Mattijs Punt, E. J. Mientjes, Audrey Vautheny, Ben Distel, Thomas Kremer, Ype Elgersma, Ravi Jagasia

**Affiliations:** 1 Neuroscience and Rare Diseases Discovery & Translational Area, Basel, Switzerland; 2 pRED Informatics Roche Innovation Center Basel, F. Hoffmann-La Roche Ltd., Basel, Switzerland; 3 Department of Clinical Genetics and Department of Neuroscience, The ENCORE Expertise Center for Neurodevelopmental Disorders, Erasmus MC, Rotterdam, The Netherlands; 4 Department of Medical Biochemistry, Amsterdam UMC, University of Amsterdam, Amsterdam, The Netherlands

**Keywords:** Biochemistry, Predictive markers

## Abstract

Angelman syndrome (AS) is a severe neurodevelopmental disorder caused by the loss of neuronal E3 ligase UBE3A. Restoring UBE3A levels is a potential disease-modifying therapy for AS and has recently entered clinical trials. There is paucity of data regarding the molecular changes downstream of UBE3A hampering elucidation of disease therapeutics and biomarkers. Notably, UBE3A plays an important role in the nucleus but its targets have yet to be elucidated. Using proteomics, we assessed changes during postnatal cortical development in an AS mouse model. Pathway analysis revealed dysregulation of proteasomal and tRNA synthetase pathways at all postnatal brain developmental stages, while synaptic proteins were altered in adults. We confirmed pathway alterations in an adult AS rat model across multiple brain regions and highlighted region-specific differences. UBE3A reinstatement in AS model mice resulted in near complete and partial rescue of the proteome alterations in adolescence and adults, respectively, supporting the notion that restoration of UBE3A expression provides a promising therapeutic option. We show that the nuclear enriched transketolase (TKT), one of the most abundantly altered proteins, is a novel direct UBE3A substrate and is elevated in the neuronal nucleus of rat brains and human iPSC-derived neurons. Taken together, our study provides a comprehensive map of UBE3A-driven proteome remodeling in AS across development and species, and corroborates an early UBE3A reinstatement as a viable therapeutic option. To support future disease and biomarker research, we present an accessible large-scale multi-species proteomic resource for the AS community (https://www.angelman-proteome-project.org/).

## Introduction

Angelman syndrome (AS) is a rare neurodevelopmental disorder characterized by severe cognitive deficits, absence of speech, sleep disturbances, seizures, motor deficits, and a generally happy demeanor [1]. The major neurological phenotypes in AS patients are driven by loss of expression of neuronal UBE3A, a HECT domain E3 ligase. During normal postnatal development, the paternal UBE3A allele in neurons is silenced by expression of a long noncoding RNA (*UBE3A-ATS*), while the maternal copy remains functional. In most AS cases, genetic defects of the maternal allele led to a complete UBE3A loss of function in neurons [2]. Rodents harboring loss of the maternal UBE3A allele have been extensively studied to model the disease. AS mouse models exhibit abnormal neurological as well as behavioral features, and thus provide a useful tool to study the molecular mechanisms underlying UBE3A-dependent neuronal dysfunction (reviewed in Rotaru et al.) [3]. Reinstating UBE3A expression via a tamoxifen-inducible cre-system in AS mice demonstrated that the rescue of behavioral abnormalities is more efficacious at early postnatal stages than in adult mice [4, 5]. Interfering with the paternal *UBE3A-ATS* transcription can reinstate expression of the paternal *UBE3A* allele to comparable level as maternal *UBE3A* [6–8]. These studies have been instrumental to the development of therapeutic strategies in AS focused on increasing neuronal UBE3A expression levels, for example by un-silencing the paternal allele using antisense-oligonucleotides (ASOs) [9, 10]. Multiple clinical trials for ASOs targeting the *UBE3A-ATS* are currently underway (ClinicalTrials.gov Identifiers: NCT04259281, NCT04428281).

Thus far, many studies investigating UBE3A substrates and their link to neuronal dysfunction in AS have identified proteins involved in neuronal function and synaptic transmission pathways, such as the GABA transporter GAT1 [11], the retrovirus-like RNA binding protein PEG10 [12], potassium channel SK2 [13], and mTORC1 activator p18/LAMTOR1 [14]. In addition, UBE3A directly regulates the abundance of several proteasomal subunits and proteasomal accessory proteins, further aggravating proteome disturbances downstream of direct UBE3A substrates [15]. Despite these efforts, the molecular components that underlie behavioral changes in AS over the course of development have yet to be resolved. For example, recent data indicates that UBE3A has a significant nuclear localization in both mice and humans in an isoform-specific manner, and that loss of nuclear UBE3A results in AS phenotypes in mice and patients [16, 17]. Most importantly, it is unclear whether UBE3A reinstatement can reverse global changes to the proteome and whether this corresponds to rescue of behavioral abnormalities [4, 5]. To this end, we provide a detailed characterization of UBE3A-dependent protein changes during brain development, across different brain regions, across species, and upon rescue of UBE3A expression, which provides insight in the underlying disease mechanism, supports the rationale for UBE3A-targeted therapeutics, and identifies putative translational biomarkers for such studies.

## Results

### AS model mice exhibit proteomic alterations at birth, which exacerbate through postnatal development

**Fig. 1:**
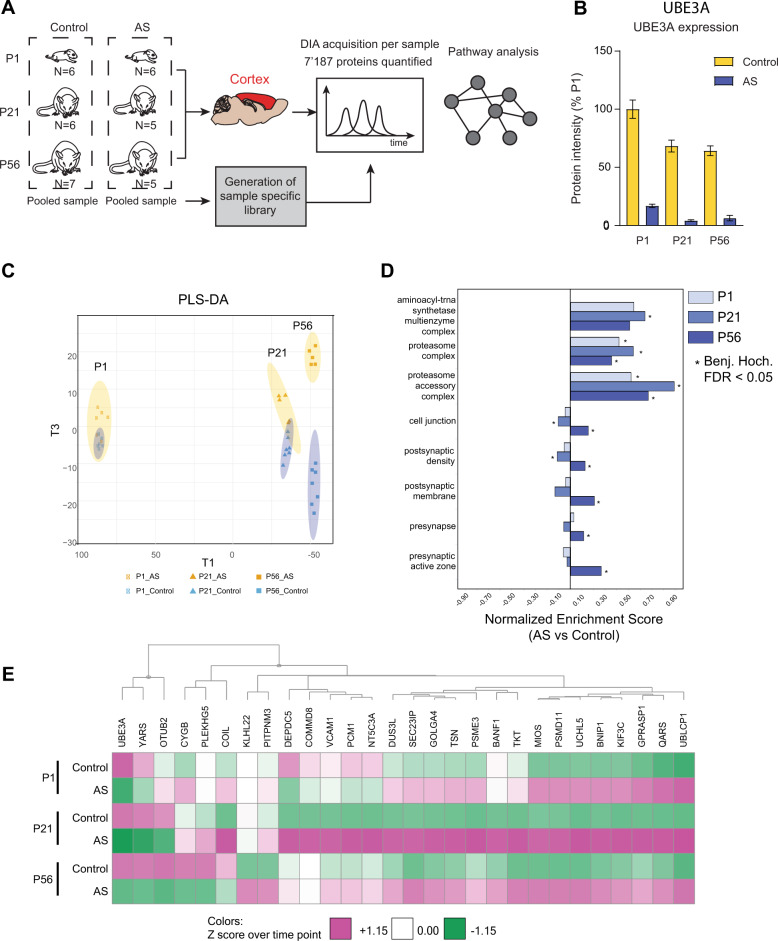
AS model mice exhibit proteomic alterations at birth, which exacerbate into adolescence and adulthood. **A** Schematic representation of experimental design. Control and AS model mice were sacrificed at P1, P21, and P56. Pooled cortical tissue of control and AS animals was used to generate a sample-specific spectral library for data-independent acquisition (DIA) mass spectrometry. Individual samples were run in DIA mode and data analyzed using the sample-specific library. Protein expression data was subjected to statistical and pathway enrichment analysis. **B** UBE3A raw protein intensity plot of cortices of control and AS model mice at P1, P21, and P56 plotted as percentage of P1 control protein levels (mean ± s.e.m. *n* = 5–6). **C** Partial least square-discriminant analysis (PLS-DA) performed on the total proteome of control and AS mouse cortices resolved according to age (T1; P1, P21, and P56) and genotype (T3; control and AS). **D** Pathway enrichment plot depicting normalized enrichment scores using 1D annotation function using GO:Cellular component genesets in AS vs. control mice. Average Z-score heatmap per time point of significantly altered (*p* value < 0.05) proteins in AS vs. control mice. Clusters are defined using Euclidean distance based on the UPGMA method. **E** A heatmap of all proteins found to be statistically significantly differentially expressed between AS and control mice in at least one time point (*q* value ≤ 0.05). Proteins are clustered using hierarchical clustering.

Data-independent acquisition (DIA) mass spectrometry-based proteomics has emerged as the method of choice for label-free quantitative proteomics, due to its increased reproducibility, depth of coverage, and high dynamic range compared to classical data dependent acquisition (DDA) [18, 19]. We chose DIA to quantify proteomic changes over the course of mouse cortical development at postnatal day P1, P21, and P56 from control and AS model mice. A pooled sample of control and AS cortices from all time points was generated, fractionated and measured in DDA mode, leading to a sample-specific spectral library containing 8270 proteins (77,439 unique peptides). Next, individual samples were measured in DIA mode and DIA data was analyzed using the sample-specific library to quantify 7187 proteins across all samples (Supplementary Table 1). Protein level data was then subjected to differential protein expression profiling analysis at each time point and pathway enrichment analysis was performed (Fig. 1A, Supplementary Tables 2, 3). Observed median biological percent coefficient of variations (% Cov) for proteins in each condition was between 11 and 15% consistent with reports in literature about DIA [18] (Fig. S1).

UBE3A protein was significantly reduced (<20% of control levels, adj. *p* value < 0.05) in AS compared to control mice at all time points. UBE3A expression levels reduced from P1 to P56 in both controls and AS, consistent with the observation that full silencing of the paternal UBE3A allele in neurons occurs during postnatal development [20] (Fig. 1B). Partial least squares discriminant analysis of all proteins (PLS-DA) separated the samples by both age (T1) and genotype (T3), with P21 and P56 being significantly different from P1. Separation along T3 revealed that AS model mice progressively diverge from control mice in terms of their proteomic profiles, with the largest differences in the adult brain (P56) (Fig. 1C).

Pathway enrichment analysis on all proteins using GO:Cellular component (GO:CC) revealed an upregulation of the aminoacyl-tRNA synthetases and proteasome complex at all postnatal stages, while synaptic pathways were significantly altered at P56, coincident with synaptic maturation (Fig. 1D). Next, we aimed to identify proteins that are differentially regulated at each developmental stage and to observe their differences over time. Altogether 28 proteins (2 at p1, 20 at p21, and 12 at p56) (Fig. 1E) passed false discovery rate (FDR) adjusted statistical significance (*q* value < 0.05) between control and AS model mice for at least one time point (Supplementary Table 2). Hierarchical clustering of these proteins revealed a genotype and developmental stage dependent effect which proteins both co-regulated and inversely regulated with respect to UBE3A expression (Figs. 1E,  S2).

Interestingly, abundance of proteins belonging to the aminoacyl-tRNA synthetase pathway (QARS, DUS3L, YARS) was both increased and decreased in AS at different time points, while proteasomal subunits, UBLCP1, UCHL5, PSME3 were increased at all time points, warranting further investigation of these pathways.

### Developmentally regulated pathway alterations in aminoacyl tRNA synthetases, proteasome, and synapse in AS model mice

**Fig. 2:**
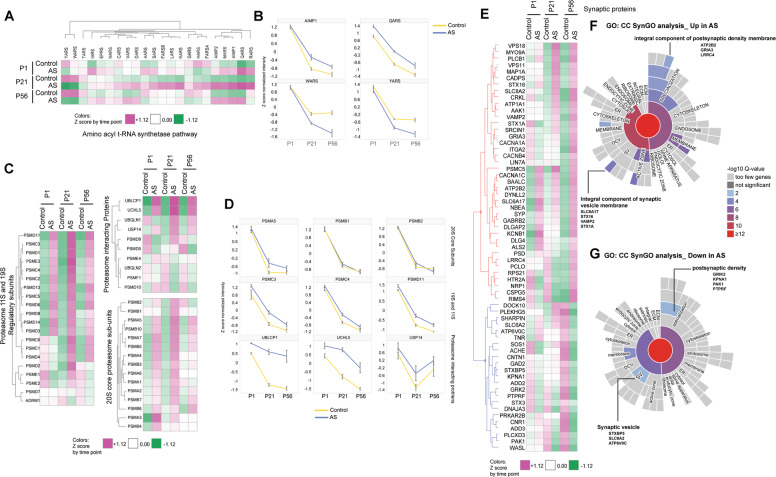
Pathway alterations in aminoacyl tRNA synthetases, proteasome and synapse are developmentally regulated. **A** Average Z-scored heatmap per time point for aminoacyl tRNA synthetases multienzyme complex and aminoacyl tRNA synthetases. Clusters are defined using Euclidian distance based on the UPGMA method. **B** Time course expression of selected proteins from the aminoacyl tRNA synthetases pathway depicting increased proteins (AIMP1, MARS) and decreased proteins (YARS, WARS) in AS vs. controls. Values represent Z-scored values. Error bars: s.e.m. (* indicates time point where the protein q value < 0.05). **C** Average Z-score heatmap per time point for proteasome complexes based on their subunit classification. Clusters are defined using Euclidian distance based on the UPGMA method. **D** Time course expression of selected proteins from the proteasome pathway depicting proteins with elevated levels from the 20S core proteasome subunits (PSMA5, PSMB1, PSMB2), 19S proteasome regulatory subunit (PSMC3, PSMC4 AND PSMD11) and proteasome interacting proteins (UBLCP1, UCHL5, USP14) in AS vs. controls. Values represent Z-scored values. Error bars: s.e.m. (* indicates time point where the protein *q* value < 0.05). **E** Average Z-scored heatmap per time point for proteins belonging to the term synapse that were significantly altered (*p* value < 0.05) at time point P56. Clusters are defined using Euclidean distance based on the UPGMA method. Clustering indicates a split between proteins with increased levels in AS (red cluster) and those with decreased levels in AS (blue cluster). **F**, **G** Sunburst visualization for proteins with increased levels (**F**) or decreased levels in AS vs. control (**G**). The genes are annotated against SynGO CC (SynGO). Colors in the sunburst plot represent enrichment *q* value scores of the UP (red) or DOWN (blue) set versus the entire measurable proteome (7126 proteins) as background. Proteins belonging to the furthest edge from the central synaptic term are labeled.

We next examined the trajectory of individual proteins within these altered pathways. These genes are expressed as early as E14.5 in wild-type mouse brain neurons, suggesting their expression prior to the onset of paternal UBE3A imprinting (Fig. S3) [20, 21]. Aminoacyl-tRNA synthetases (ARS) are evolutionarily conserved enzymes involved in the ligation of amino acids to their cognate tRNAs and occur either free or as part of the ARS multienzyme complex (MSC) [22]. We examined the expression of all ARS and MSC proteins across brain maturation (Fig. 2A), which revealed that Class I ARS proteins, specifically MARS, QARS, RARS, AIMP1, and AIMP2, which belong to the MSC, are increased in AS. Conversely, ARS proteins involved in aromatic amino acids loading, namely tryptophanyl-tRNA synthetase WARS, and tyrosyl-tRNA synthetase YARS, were decreased in AS (Fig. 2A). Interestingly, irrespective of genotype there is a strong reduction of these proteins during postnatal brain development, with disease alteration diverging dramatically at P21 and P56 in AS (Fig. 2B).

Proteins corresponding to all components of the proteasome were altered from birth and persistent to adulthood. We observed a consistent increase in abundance of several proteasome interacting proteins (UCHL5, UBLCP1, USP14), as well as of proteins belonging to the 11S and 19S regulatory subunits [23] (PSMD11, PSMC3, PSMD1, PSME3), while the changes in the 20S core proteasome subunits followed a similar trend but were comparably minor (Fig. 2C, D).

Next, we examined synaptic proteins that were selectively altered at P56 in AS model mice (AS vs. control, un-adj. *p* value < 0.05) and found 63 proteins that fulfilled these criteria (Fig. 2E). Heatmap visualization and hierarchical clustering of these proteins revealed a bi-directional AS genotype effect, with sets of proteins showing increased levels (red cluster, 40 proteins) and decreased levels (blue cluster, 23 proteins) in AS model mice compared to controls. Synapse GO [24] (SynGO CC) enrichment analysis for the set of 40 proteins elevated compared to controls showed that they distributed on both pre- and postsynaptic sites. Proteins belonging to synaptic vesicles and presynaptic active zone (STX1A, SYP, VAMP2), as well as proteins that are integral components of the postsynaptic density (GRIA3, ATP2B2, LRRC2) were significantly enriched (Fig. 2F, top; Supplementary Table 3). SynGO analysis for the set of 23 proteins which are decreased in AS compared to controls similarly revealed proteins that belong to both pre- and postsynaptic compartments, with synaptic vesicle proteins (STXBP5, SLC6A2, ATP6V0C) and postsynaptic density proteins (GRK2, KPNA1, PAK1, PTPRF) being decreased in AS at P56 (Fig. 2G).

Thus, protein alterations in AS model mice are dynamic; ARS and proteasome subunits are altered from birth, while many changes in synaptic proteins develop over final stages of brain maturation.

### Adult AS model rats (P84) recapitulate the proteomic alterations observed in AS model mice across different brain regions

**Fig. 3:**
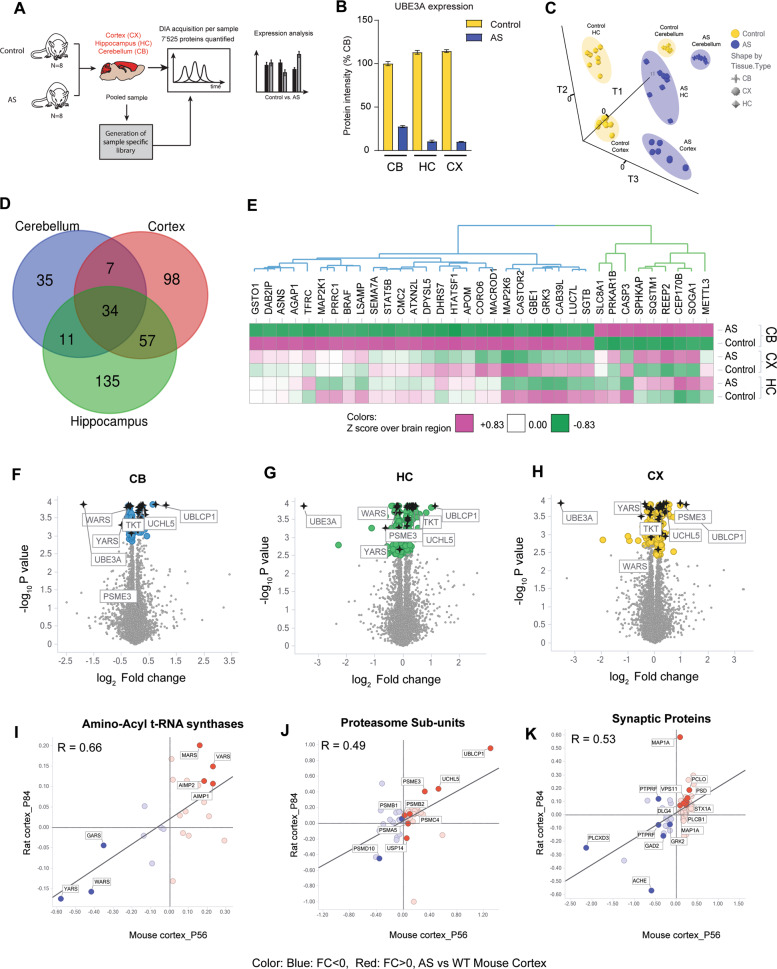
Adult AS model rats (P84) recapitulate the proteomic alterations observed in AS model mice across different brain regions. **A** Schematic representation of experimental design. Control and AS model rats were sacrificed at P84. Pooled tissue of cerebellum (CB), cortex (CX), and hippocampus (HC) of control and AS animals was used to generate a sample-specific spectral library in DDA (data dependent acquisition) mode. Individual samples were further analyzed using data-independent acquisition (DIA) mass spectrometry. **B** UBE3A raw protein intensity plot of cerebellum (CB), cortex (CX), and hippocampus (HC) of control and AS model rats plotted as percentage of CB control protein levels (mean ± s.e.m.). **C** Partial least square-discriminant analysis (PLS-DA) performed on the total proteome of control and AS model rats resolved according to brain region (T1 and T2; cerebellum, cortex, hippocampus) and genotype (T3; control and AS), and projected in 3D space. **D** Venn diagram of statistically significant (adj. *p* value < 0.05) proteins altered in each brain region in AS model rats. **E** Heatmap of proteins that pass statistical significance in the cerebellum. Proteins fall into two categories: increased (green cluster) or decreased (blue cluster) levels in AS compared to controls. **F**–**H** Volcano plot of *p* value vs. Log_2_ fold change per brain region. Proteins that are statistically significant in each pairwise comparison are highlighted (Blue: CB, Green: HC, Yellow: Cortex). Proteins significant in all three brain regions are marked in black stars. Subset of proteins of interest from Fig. 1E is labeled. **I**–**K** Log_2_ fold change correlation plots between mouse cortex and rat cortex for proteins in the aminoacyl tRNA synthetases pathway (**G**), proteasome subunits (**H**), and synaptic proteins (**I**) as filtered in Fig. 2F. Correlation coefficients are calculated using Pearson’s method.

We next explored if the observed alterations are conserved across species and brain regions by making use of a newly available AS rat model [25]. We performed DIA based LC-MS/MS analysis of three rat brain regions (CB: Cerebellum, HC: Hippocampus, and CX: Cortex) in adult (P84) control and AS model rats (Fig. 3A, Supplementary Table 4). A hybrid spectral library was generated from DDA runs on a fractionated pooled sample of all three brain regions from all samples combined with DirectDIA measurements to generate a library of 8928 proteins (116,603 unique peptides), allowing us to quantify 7525 proteins across all samples, with coefficients of variation around 10% per sample type (Fig. S4). UBE3A expression was robustly reduced in all three brain regions in AS model rats (Fig. 3B), similar to AS model mice [26, 27]. UBE3A levels in the cerebellum of control rats were lower compared to hippocampus and cortex, while residual UBE3A levels in the AS model rat cerebellum were higher (27% of control levels in cerebellum compared to <10% of control levels in hippocampus and cortex; Fig. 3B). Subsequent PLS-DA analysis revealed a robust separation between genotypes (T3) and brain regions (T2, T1) (Fig. 3C). The proteomic profile of the cerebellum was distinct to the cortex and hippocampus, likely reflecting different developmental origin and cyto-architecture.

Examination of the proteins significantly altered in the brains of AS model rats revealed that 34 proteins were significantly changed across all three brain regions (*q* value < 0.05) and that there was large overlap in the alterations in the hippocampus and cortex (Fig. 3D, Supplementary Table 5). Significant hits in the cerebellum were visualized by hierarchical clustering, revealing sets of proteins with increased (green cluster) and decreased (blue cluster) levels compared to controls (Figs. 3E, S5). Interestingly, several proteins (including GSTO1, DAB2IP, ASNS, AGAP1), were specifically altered in the cerebellum in AS, suggestive of cerebellar neuron-specific regulation of these substrates (Fig. S6A). Proteins belonging to proteasomal subunits (including UBLCP1, UCHL5, PSME3) and the ARS proteins YARS and WARS were altered in all three brain regions (Fig. 3F–H, black stars).

Next, we examined the correlation between altered proteins corresponding to the 3 major pathways in AS P56 mouse and P84 rat cortices (Fig. 3I–K), and across rat brain regions (Fig. S6B–G). We divided each pathway into sets of proteins that are increased (in red) or decreased (in blue) in AS model mice cortex at P56 compared to controls. Correlation plots of fold changes for the ARS pathway revealed a significant correlation between rat and mice (*R* = 0.66, *p* = 4.29E−004), with YARS, WARS and GARS decreased, and MARS, VARS, AIMP1 and AIMP2 increased in AS compared to controls in both mice and rats (Figs. 3I, S5A). In addition, protein changes for these pathways were seen across rat cortex, hippocampus and, to a lesser extent, with cerebellum (rat cortex to cerebellum: *R* = 0.79, *p* = 4.42E−006; Fig. S6B; rat cortex to hippocampus: *R* = 0.93, *p* = 8.84E−011, Fig. S6C). Proteasome complex proteins showed a robust correlation between rat and mouse cortices (Fig. 3J) (*R* = 0.49, *P* = 2.12E−004) and across brain regions (Fig. S5B). UBLCP1, UCHL5, and PSME3 were consistently elevated in adult rats across all three brain regions (Figs. 3J,  S5B,  S6D, E). Protein levels of proteasome assembly chaperone 3; PSME3 and 26S proteasome non-ATPase regulatory subunit 10; PSMD10 were consistently reduced in AS compared to controls in AS model mice as well as in AS rat brain regions. Like for the ARS pathway, cortex and hippocampus showed more similarity to each other while the cerebellum diverged slightly (Fig. S6D, E). Analysis of synaptic protein alterations showed both similarities and differences between species and across rat brain regions (Figs. 3K,  S5C,  S6F, G). AS rat cortex was similar to both mouse cortex (*R* = 0.53, *P* = 4.86E−006), and rat hippocampus (*R* = 0.56, *P* = 1.59E−006), while diverging in AS cerebellum (*R* = 0.21, *P* = 8.60E−002). Of interest, the synaptic proteins GAD2, ACHE, PLCXD3, and GRK2, were decreased in AS in all brain regions and across species, while PSD, DLG4, PLCB1, and VPS11, were increased in AS compared to controls. Others such as MAP1A, PCLO, PTPRF, STX1A, showed brain region- or species-specific differences (Fig. S5C).

### Reinstatement of UBE3A expression in juvenile and adult AS model mice rescues altered proteomic state to varying degrees

**Fig. 4:**
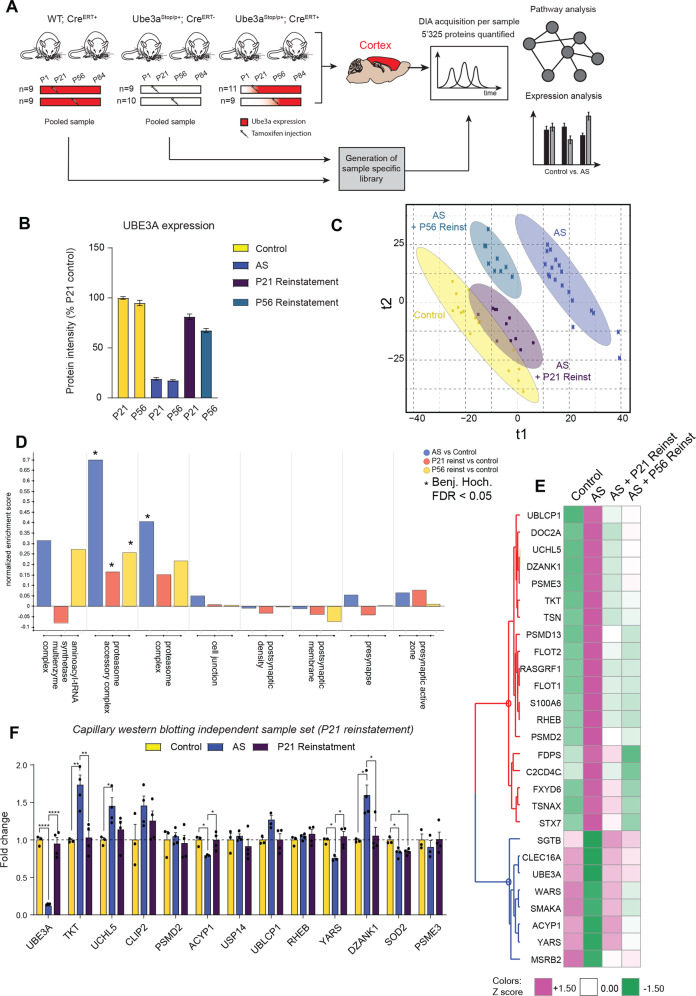
Reinstatement of UBEA in both juvenile and adolescent AS model mice rescues protein and pathway alterations. **A** Schematic representation of experimental design. Control mice (WT; Cre^ERT2+^), AS model mice (Ube3a^Stop/+^; Cre^ERT2−^), and mice with UBE3A reinstatement (Ube3a^Stop/+^; Cre^ERT2+^) were injected with tamoxifen at P21 or P56 and sacrificed at P84. Cortical tissue of both control and AS model mice was pooled to generate a sample-specific spectral library in data-dependent acquisition (DDA) mode. Individual samples were further analyzed in data-independent acquisition (DIA) mode. **B** UBE3A raw protein intensity plot in cortices of P21 and P56 injected groups of control mice, AS model mice, and mice with UBE3A reinstatement, plotted as percentage of P21 control protein levels (mean ± s.e.m.). **C** Partial least square-discriminant analysis (PLS-DA) performed on the total proteome of control and AS mouse cortices resolved according to time point of UBE3A reinstatement (T1; P21 and P56) and genotype (T2; control, AS, and reinstatement). **D** Pathway enrichment plot depicting normalized enrichment scores using 1D annotation function using GO:Cellular component genesets in AS vs. control mice (blue), AS model mice with Ube3a reinstatement at P21 vs. control (red) and AS model mice with UBE3A reinstatement at P56 vs. control (yellow). Select pathways are visualized as observed in Fig. 1D. **E** Heatmap of significantly altered hits between any of the four conditions using ANOVA (adj. *p* value < 0.05). Colors represent average Z-scored protein intensities for each protein. **F** Orthogonal validation of UBE3A targets in an independent sample set of control mice (*N* = 3), AS model mice (*N* = 4), and mice with UBE3A reinstatement at P21 (*N* = 4) with capillary western blotting. Statistical analysis was performed using one-way ANOVA followed by Tukey’s post hoc test. (*p < 0.05; **p < 0.01; ****p < 0.0001).

To explore if these alterations in potential UBE3A targets and pathways could be rescued by UBE3A reinstatement during clinically relevant therapeutic time points, i.e., in juvenile and adult mice, we utilized an AS mouse model harboring a tamoxifen-inducible UBE3A allele [4]. Control (WT; Cre^ERT2+^), AS (Ube3a^Stop/p+^), and UBE3A reinstatement mice (Ube3a^Stop/p+^; Cre^ERT2+^) were injected with tamoxifen at either P21 or P56, corresponding to juvenile and adult developmental stages, and sacrificed at P84 to compare rescue at the two time points (Fig. 4A). Cortical tissue was used to quantify 5′325 proteins across all samples in DIA mode and analyzed with pooled libraries created from both control and AS groups (Supplementary Table 6).

UBE3A reinstatement at P21 and P56 was able to rescue cortical UBE3A protein levels from <20 to 81 and 71% of that of control animals, respectively, as measured by LC/MS/MS (Fig. 4B). PLS-DA separated the samples by both time point of reinstatement (T1) and genotype (T2), with control being significantly different from AS model mice (Fig. 4C) with UBE3A rescue at P21 showing a near complete reversal, while P56 reinstatement partially rescued proteomic alterations.

We next performed expression and pathway analysis of differentially changed proteins to map trajectories of UBE3A downstream targets between different groups (Supplementary Tables 7, 8). Pathway enrichment analysis on all proteins using GO:Cellular component (GO:CC) analysis comparing AS with control, or AS with reinstatement at P21 or P56, revealed a rescue of ARS at P21 but not P56, and rescue of both the proteasome complex and synaptic protein genesets at both time points (Fig. 4D).

A heatmap of the top 27 individual proteins that were significantly altered between any of the four sample sets reveals that the majority of proteins could be reverted to some extent at both time points (*q* value < 0.05; Fig. 4E). While the majority of proteins, including top candidates belonging to ARS (such as YARS and WARS), and proteasomal subunits or proteasome accessory proteins (e.g., UBLCP1, UCHL5, PSME3), show an enhanced rescue at P21 vs. P56; the expression of several synaptic proteins including FDPS, C2CD4C, FXYD6, TSNAX, and STX7 were normalized at P56, indicating that their response to UBE3A can vary.

To confirm a subset of the top identified UBE3A targets with an orthogonal method, we performed capillary western blotting from an independent cohort of control, AS, and P21 reinstatement mice (Fig. 4F) which confirmed changes and rescue of TKT, UCHL5, ACYP1, YARS, DZANK, and SOD2. qPCR analysis of these hits showed no significant changes, indicating that changes in expression levels are at translational or post-translational level (Fig. S7).

### Transketolase is a novel target of UBE3A in rodents and humans

**Fig. 5:**
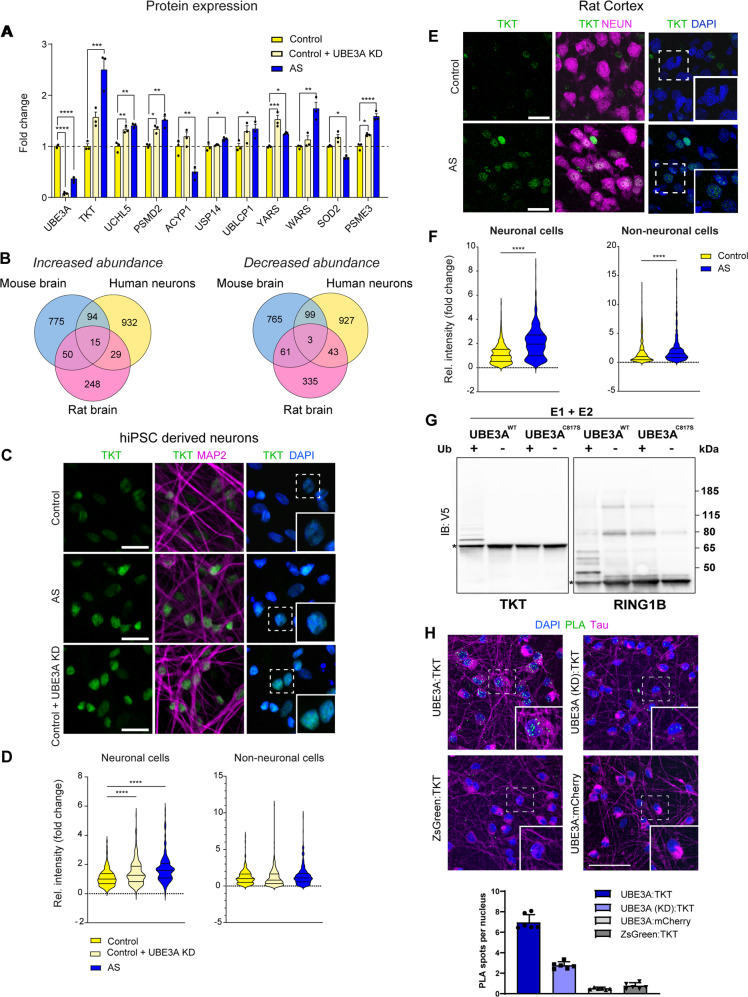
Transketolase is a novel target of UBE3A deregulated in rodent and human AS disease models. **A** Capillary western blot analysis of UBE3A targets in control, control + UBE3A KD ASO, and AS lines of hiPSC-derived neurons. *N* = 3 for all samples. Statistical testing was performed using one-way ANOVA followed by Tukey’s post hoc test. (*p < 0.05; **p < 0.01; ***p < 0.001; ****p < 0.0001). **B** Venn diagrams showing proteins shared between species with increased (fold change > 0.25 log2) or decreased (fold change < −0.25 log2) protein expression in AS condition compared to controls. **C** Immunocytochemical images of transketolase (TKT) and the neuronal marker MAP2 in hiPSC-derived neurons of control, control + UBE3A KD ASO, and AS lines. Nuclei were counterstained with DAPI. Scale bars: 25 μm. **D** Quantification of nuclear TKT signal in neurons (MAP2-positive) and non-neuronal cells (MAP2-negative). Individual data points from 8 images taken from different wells in two independent experiments (neuronal differentiation and ASO treatment) were plotted. Statistical analysis was performed using Kruskal–Wallis test followed by Dunn’s post hoc test. **E** Immunohistochemical images of transketolase (TKT) and the neuronal marker NEUN in the primary visual cortex of adult control and AS model rats. Nuclei were counterstained with DAPI. Scale bars: 25 μm. **F** Quantification of nuclear TKT signals in neurons (NEUN-positive) and non-neuronal cells (NEUN-negative). Individual data points from three images per animal were plotted (control: *N* = 3, AS: *N* = 2 animals). Statistical analysis was performed using Kruskal–Wallis test followed by Dunn’s post hoc test. **G** Bacterial ubiquitination assay for TKT and RING1B. Representative data from two independent experiments. **H** Proximity ligation assay of UBE3A with TKT upon treatment with NT ASO (UBE3A:TKT), UBE3A with TKT upon UBE3A knockdown (UBE3A (KD):TKT), and the two non-interacting controls UBE3A with mCherry (UBE3A:mCherry), and TKT with Zs-Green (TKT:Zs-Green). For each pair, six wells with 5′000–10′000 nuclei were analyzed. Scale bars: 50 μm.

We next investigated whether the top UBE3A-dependent candidates identified in mice and upon UBE3A reinstatement are altered in induced pluripotent stem cells (iPSCs) derived AS patient neurons [12, 28, 29] using capillary western blotting using our established antibody panel (Fig. 5A). All proteins tested showed significant upregulation in AS neurons compared to controls, including all of those associated with the proteasome (UCHL5, PSMD2, USP14, UBLCP1, PSME3), and the ARS pathway (YARS, WARS). Furthermore, treatment of control neurons with an UBE3A-targeting ASO to lower UBE3A expression (UBE3A KD) phenocopied the changes seen in AS patient neurons, albeit with smaller fold changes in most cases, suggesting UBE3A dependence (Fig. 5A).

To identify in a more systematic way additional proteins that are changed between species in an UBE3A-dependent manner, we calculated the overlap of increased (fold change > 0.25 log2) or decreased (fold change < −0.25 log2) protein abundance in the AS condition compared to controls in mouse, rat, and human patient neurons from a previous study [12]. We found 15 proteins altered across species with increased abundance, including IQGAP2; GPRASP1; ZEB2; YTHDC2; GIGYF1; RGS3; PARG; MIOS; WDR59; PRMT9; CCDC120; TKT; KLHDC4; UBLCP1; HIST1H1B, and three proteins with decreased abundance in AS across all species: UBE3A; GOLIM4; NFYA (Fig. 5B).

Consistently, across AS patient neurons, and AS mouse and rat brains, TKT was robustly altered. Distinct from other enzymes in the pentose phosphate pathway, TKT has been previously reported to be enriched in the nucleus of non-neuronal cells, and to contain a nuclear localization signal [30]. Similarly, TKT appeared to have pronounced nuclear enrichment in human and rat neurons (Fig. 5C, E). In human neurons, co-labeling with DAPI and MAP2 revealed that TKT expression was higher in the nuclei of neurons compared to non-neuronal cells (MAP2+ vs. MAP2− cells) and increased in AS neurons in a UBE3A-dependent manner (Fig. 5C, D). TKT nuclear intensity in both neuronal iPSC-derived AS and UBE3A KD cultures revealed significant upregulation of TKT expression (MAP2-positive nuclei compared to controls, +59%; Figs. 5C, D,  S8). Consistent with the patient neurons, nuclear TKT signal was significantly increased in the AS rat cortexes (+95%; Figs. 5E, F, S8). In rat brains, there was a small increase in non-neuronal cells, which could be due to UBE3A gene dosage reduction (Fig. 5F).

To assess if TKT is a direct target of UBE3A, we used a previously described cellular ubiquitination assay performed in *E. coli* [17, 31]. To this end, *E. coli* cells were transformed with plasmids encoding UBE3A (or the catalytically inactive variant) and other components needed for ubiquitination, ubiquitin, and TKT or RING1B. RING1B is a well-established target of UBE3A and serves as positive control [32]. The presence of active UBE3A and all components of the ubiquitination cascade leads to formation of slower migrating bands indicative of ubiquitination, for both RING1B and TKT, but not in absence of UBE3A or with the catalytically inactive variant UBE3A^C817S^ (Fig. 5G). This strongly suggest that UBE3A can directly ubiquitinate TKT. To test whether TKT and UBE3A interact in human neurons, we performed proximity ligation assay (PLA) on commercially available human iCell GlutaNeurons (FUJIFILM Cellular Dynamics). Neurons were treated for 7 days with either non-targeting or UBE3A KD ASOs before neurons were stained for Tau, and the UBE3A:TKT interaction with Duolink^®^ In Situ Green Link (Sigma) labeled specific antibodies. PLA signal was located in both cytoplasm and nuclei of neurons, and nuclear signal was significantly decreased upon treatment with UBE3A KD ASO compared to NT controls (Fig. 5H). To confirm specificity of both antibodies, TKT:Zs-Green and UBE3A:mCherry were included as non-interacting pairs (Fig. 5H).

UBE3A has been shown to be expressed in several isoforms that differ from each other in cellular localization. In humans, three functional isoforms exist that vary at the N-terminus [33, 34], while in mice, there are two isoforms, a shorter, predominantly nuclear isoform (mUBE3A-Iso3), and a longer, cytosolic isoform (mUBE3a-Iso2). Loss of the nuclear UBE3A isoform has been shown to be sufficient to induce AS phenotypes in mice [16]. Given the UBE3A-dependent upregulation of TKT in neuronal nuclei, we asked whether its regulation is UBE3A isoform-specific. Lysates from cortical tissue of mUBE3A-Iso3 knockout mice and control mice were analyzed with capillary western blotting. Compared to wild-type controls, levels of both TKT and UCHL5 (a known UBE3A target localized to the nucleus) were elevated in tissue of mUBE3A-Iso3 knockout mice (+12.8% and +28.4%, respectively) (Fig. S9). Although more evidence is needed to confirm TKT as a nuclear target of UBE3A, these results suggest that TKT protein levels are at least partially controlled by the nuclear isoform mUBE3A-Iso3. Systematic evaluation of subcellular localization of disease altered proteins, revealed that a subset of proteins have nucleoplasmic localization, including UBLCP1, UCHL5, DZANK1, TKT, PSME3, see Table S9).

## Discussion

In this study, we performed a global analysis of UBE3A-driven temporal and spatial remodeling of the rodent proteome. Together with our previous proteomic analysis of AS patient derived neurons [12], we highlight disease alterations across species, demonstrate that the AS proteome exacerbates over the course of postnatal development and that it can be reverted by UBE3A reinstatement at juvenile and, to a lesser extent, in young adults. In addition, we identified three major cellular pathways affected by UBE3A loss in mice that were also observed in rat and human models of AS: the proteasome, ARS, and several synaptic pathways. These results imply that the regulation of global cellular processes is controlled by UBE3A via its direct substrates as well as a myriad of secondary downstream targets, which may impact brain development. To make these large datasets accessible to the scientific community, we constructed a rolling database containing the proteomic analyses presented in this study and combined it with datasets from previous studies done in human patient neurons, allowing comparison of individual hits across species, development and after rescue (Fig. S10, https://www.angelman-proteome-project.org/).

The most striking changes observed in the AS proteome in all species and across all developmental stages, was the upregulation of proteasomal subunits and ARS. It has been reported previously that UBE3A can directly interact with the 26S proteasome and regulate turnover of proteasomal subunits [15, 16, 35, 36]. Our data shows that changes in protein abundance affected the 19S and 11S regulatory subunit and proteasomal accessory proteins to a larger extent than the 20S core [37]. ARS are a family of nuclear-encoded enzymes that ensure correct translation by conjugating amino acids to their cognate tRNA molecule, providing a key initial step for protein translation [22], some of whom have been identified as UBE3A binders [38]. A range of nervous system disorders are linked to mutations in ARS proteins, and rare genetic forms of amino acid deficiencies result in autism [39, 40]. Interestingly, individual proteins in the ARS pathway are not universally elevated; specifically, QARS and AIMP1 proteins were increased, while YARS and WARS were decreased in AS model mice, which may suggest a more complex UBE3A-dependent regulatory mechanism comprising both direct and indirect effects. It is conceivable that UBE3A loss causes proteosome dysfunction and correspondingly alterations in the protein translation machinery, which will lead to cumulative effects on the AS proteome. Future work will be required to determine the precise role of the proteasome dysfunction on AS proteome remodeling. In contrast to proteasome and ARS pathways, synaptic candidates were mostly altered at later developmental stages coincident with the full maturation of the nervous system. While synaptic protein changes were observed at both P21 and P56, the UBE3A-dependent alterations were more pronounced at P56. Our analysis revealed that candidates localized to both pre- and postsynaptic compartments, including integral components of synaptic vesicles, presynaptic active zone, and postsynaptic density, indicating overall synaptic perturbations rather than changes in a single synaptic compartment. Future studies should address to what extent UBE3A-dependent proteasomal and dysregulation of ARS contributes to the disease and to indirect downstream molecular changes, for instance in proteins of the synapse in AS.

Our study represents the first proteomic study of AS rat brain tissue [25], in which we confirmed top candidates and dysregulation of the proteasome and the ARS proteins identified in AS model mice This includes members of the ARS family YARS and WARS, proteasomal subunits, accessory proteins such as UBLCP1 and UCHL5, and the metabolic enzyme TKT, which showed consistent upregulation across all our AS datasets. Individual brain region analysis in rats identified commonality and regional differences between cortex, hippocampus, and cerebellum, areas that show functional impairment in AS patients and have been the primary focus of research over the years [41]. The cerebellar AS proteome diverged from the cortex and hippocampus, which might have implications in motor dysfunction and ataxia seen in rodent models and AS patients. Several of the cerebellar specific protein alterations are associated with neurological diseases including epilepsy and intellectual disability including BRAF, CRMP5 and ASNS [42]. Notably, cerebellum-specific metabolic dysfunction can result in autism-like phenotypes in mice [43], thus understanding region-specific dysfunction may be warranted.

Restoring UBE3A expression is a promising therapeutic strategy for AS currently undergoing clinical trials (ClinicalTrials.gov Identifiers: NCT04259281, NCT04428281). In a conditional AS mouse model harboring an inducible maternal UBE3A allele, age-dependent rescue of specific behavioral phenotypes was demonstrated after UBE3A reinstatement [4]. Moreover, although a single intracerebroventricular injection of an UBE3A-ATS ASO in adult AS model mice failed to rescue most behavioral phenotypes, ASO injection of newborn mice rescued many behavioral phenotypes [7, 9]. Strikingly, restoring UBE3A expression at either P21 or P56 restored proteomic homeostasis to a significant degree, albeit P21 rescue being more efficient. To a large extent, proteasome and synaptic pathway alterations were reverted at both time points, and this is in line with the observation that most electrophysiological parameters can also be restored upon adult UBE3A reinstatement [44]. These results highlight that disease trajectories of proteins and associated pathways are UBE3A-dependent and reversible.

In a previous study, proteomic analysis of patient derived iPSC neuronal cultures and after UBE3A reinstatement using ASOs revealed a set of human specific UBE3A targets, including the GAG domain containing protein PEG10 [12]. In addition, several top candidates identified in the patient neurons were also altered in AS models, including PPID, DST, and UCHL5. Several additional proteasome and ARS proteins were changed in AS neurons and phenocopied by knockdown of UBE3A in control neurons. Cross species comparison between human iPSC neurons, mouse, and rat brains revealed only a small overlap of candidates changed in the AS condition. Whilst comparisons between the human neuronal disease model with adult rodent brains has several obvious shortcomings, it is conceivable that species-specific differences could be a factor in the regulation of UBE3A substrates similar as previously reported for PEG10 [12].

Notably, TKT; a rate limiting enzyme of the pentose phosphate pathway; showed consistent cross species and UBE3A-dependent changes. Intriguingly, TKT localization in both human neurons and rat brain tissue was found to be predominantly nuclear, even though the PPP takes place in the cytosol [45]. In this study, evidence of endogenous TKT ubiquitination by UBE3A in AS mouse models and/or human iPSC-derived neurons was not possible with available TKT antibodies. PLA analysis is suggestive that UBE3A and TKT are in close vicinity in human neurons. Future experiments, such as di-Gly proteomics on AS models could shed light on direct substrates of UBE3A and also enable the identification of the exact site of TKT that is ubiquitinated by UBE3A. In addition, results from a mouse model harboring a deletion of the nuclear isoform of UBE3A [33] suggests that TKT is directly regulated by nuclear UBE3A. Non-canonical regulatory functions have been described for many metabolic enzymes, including glycolytic enzymes that can act as protein kinases and transcriptional regulators [46], and in line with this there is a report that nuclear TKT interacts with EGFR functionally independent of TKT enzymatic activity [47]. Future studies are also warranted to elucidate whether TKT has a role AS pathophysiology and whether non-canonical functions play a role. Future efforts will be required to determine the altered human proteome from AS brain postmortem samples and analysis of patient CSF samples to determine if TKT and other proteins are secreted from neurons in disease, and to validate them as downstream UBE3A biomarkers.

## Materials and methods

### Animals

Mice were housed in individually ventilated cages (IVC; 1145T cages from Techniplast) in a barrier facility. All animals were kept at 22 ± 2 °C with a 12 h dark and light cycle, and provided with mouse chow (801727CRM(P) from Special Dietary Service) and water ad libitum. All animal experiments were conducted in accordance with the European Commission Council Directive 2010/63/EU (CCD approval AVD101002016791).

### Data-independent acquisition (DIA) mass spectrometry

Total protein profiling of rat and mouse tissue was performed at Biognosys AG (Schlieren, Switzerland) using Biognosys’ Hyper Reaction Monitoring (HRM™) label-free discovery proteomics workflow.

All measurements were performed in a randomized and blinded fashion and balanced for genotype age and treatment condition at Biognosys prior to sample preparation. All.raw files will be made available on Massive server: Massive ID: MSV000087972. Proteomic analysis of angelman rodent models.

For detailed methods on sample preparation, HPRP fractionation, library generation, and acquisition, see Supplementary Methods.

### HRM data analysis

HRM mass spectrometric data were analyzed using Spectronaut Pulsar software (Biognosys, version 12 and 13.8.190930). The false discovery rate on peptide and protein level was set to 1%, data was filtered using row based extraction. The assay library (protein inventory) generated in this project combined with the ones from MCP (Bruderer et al. [48]) was used for the analysis. The HRM measurements analyzed with Spectronaut were normalized using local regression normalization [44].

For bioinformatics analyses of proteomic data see Supplementary Methods.

### Capillary western blot

The investigators were not blind to the genotypes during analysis and acquisition of capillary western blotting data. Protein expression of putative UBE3A targets in mouse brain (*n* = 3 animals per group) and hiPSC lysates (*n* = 3 independent differentiations per group) was analysed by automated capillary western blotting (Sally Sue, Protein Simple). All experimental steps were carried out according to the manufacturer’s instructions. For brief overview of experimental procedure see Supplementary Methods.

### hIPSC culture

Cell culture of NPCs and neurons was performed as described in Costa et al. and Pandya et al. [12, 28].

### IPSC stainings and imaging

The investigators were not blind to the genotypes during analysis and acquisition of imaging data. Control and AS deletion neurons were cultured in BGAA media. Media was changed 1 day before treatment. hiPSC-derived neurons were treated with 1 µM ASO in PBS for 6 weeks during neuronal differentiation. Post treatment, cells were immediately fixed and stained for TKT (Sigma-Aldrich, HPA029480; 1:200), UBE3a (Sigma-Aldrich, SAB1404508; 1:200) and Map2 (Abcam, ab5392; 1:500) and DAPI as previously described [12].

### Imaging and imaging data analysis

Images were acquired on a Leica TCS SP5 confocal microscope. Automated quantification of nuclear UBE3A and TKT expression was performed by using DAPI staining as a mask and measuring the integrated density of fluorescence in each cell (ImageJ). Manual thresholding of HuC/D staining in each independent experiment (*n* = 2 independent differentiations) was performed to distinguish neuronal from non-neuronal cells. Replicates from two independent differentiations were combined for data analysis. Statistical analysis was performed with GraphPad Prism Software (version 8) using Kruskal–Wallis test followed by Dunn’s post hoc test.

### Proximity ligation assay (PLA)

Human iCell^®^ GlutaNeurons (FUJIFILM Cellular Dynamics) were seeded according to the manufacturer’s instructions. Cells were treated for 7 days with 5 μM ASO. PLA was performed using the Duolink^®^ In Situ Green Kit (Sigma-Aldrich) according to the manufacturer’s instructions. Antibodies: anti-TKT (Proteintech, 11039-1-AP), anti-UBE3a (Sigma, SAB1404508), anti-mCherry (Cell Signaling Technology, 43590S), anti-ZsGreen1 (Origene, TA180002) and anti-Tau (2E9) Alexa Fluor^®^ 647 (Novus Biologics, NBP2-25162AF647). Images were acquired by a Perkin Elmer Opera Phenix plus high-content confocal imaging system with a 63x water objective and analysis was done using Harmony high-content analysis software 5.1. For each pair, six wells with 5′000–10′000 nuclei each were counted.

### Immunohistochemistry

Brain tissue was fixed with 4% PFA and prepared for cryosectioning. Immunohistochemical staining for Tkt (Sigma-Aldrich, HPA029480; 1:200), Ube3a (Sigma-Aldrich, SAB1404508; 1:400), and NeuN (Sigma-Aldrich, MAB377; 1/500) in rat brain tissue was performed as previously described with more details in Supplementary Methods [12].

### Imaging and data analysis

Images were acquired on a Leica TCS SP8 confocal microscope. Z-stack projections were performed using the ‘sum of slices’ command on FIJI (ImageJ). Automated quantification of nuclear UBE3A and TKT expression was performed by using DAPI staining as a mask and measuring the integrated density of fluorescence in each cell. Manual thresholding of NeuN staining was performed and kept consistent across animals to distinguish neuronal from non-neuronal cells. Statistical analysis was performed with GraphPad Prism Software (version 8) using using Kruskal–Wallis test followed by Dunn’s post hoc test.

## Supplementary information

Supplementary Table 1

Supplementary Table 2

Supplementary Table 3

Supplementary Table 4

Supplementary Table 5

Supplementary Table 6

Supplementary Table 7

Supplementary Table 8

Supplementary Table 9

Supplementary Figures

Supplementary Methods

Supplementary Table Descriptions
