# Supplementary material for: A cross-species spatiotemporal proteomic analysis identifies UBE3A-dependent signaling pathways and targets
Source: Mol Psychiatry. 2022 Mar 9;27(5):2590–601. doi: 10.1038/s41380-022-01484-z (PMC9135630; doi:10.1038/s41380-022-01484-z)
Supplement: Supplementary file 10 — Supplementary Figures [file 41380_2022_1484_MOESM10_ESM.pdf]

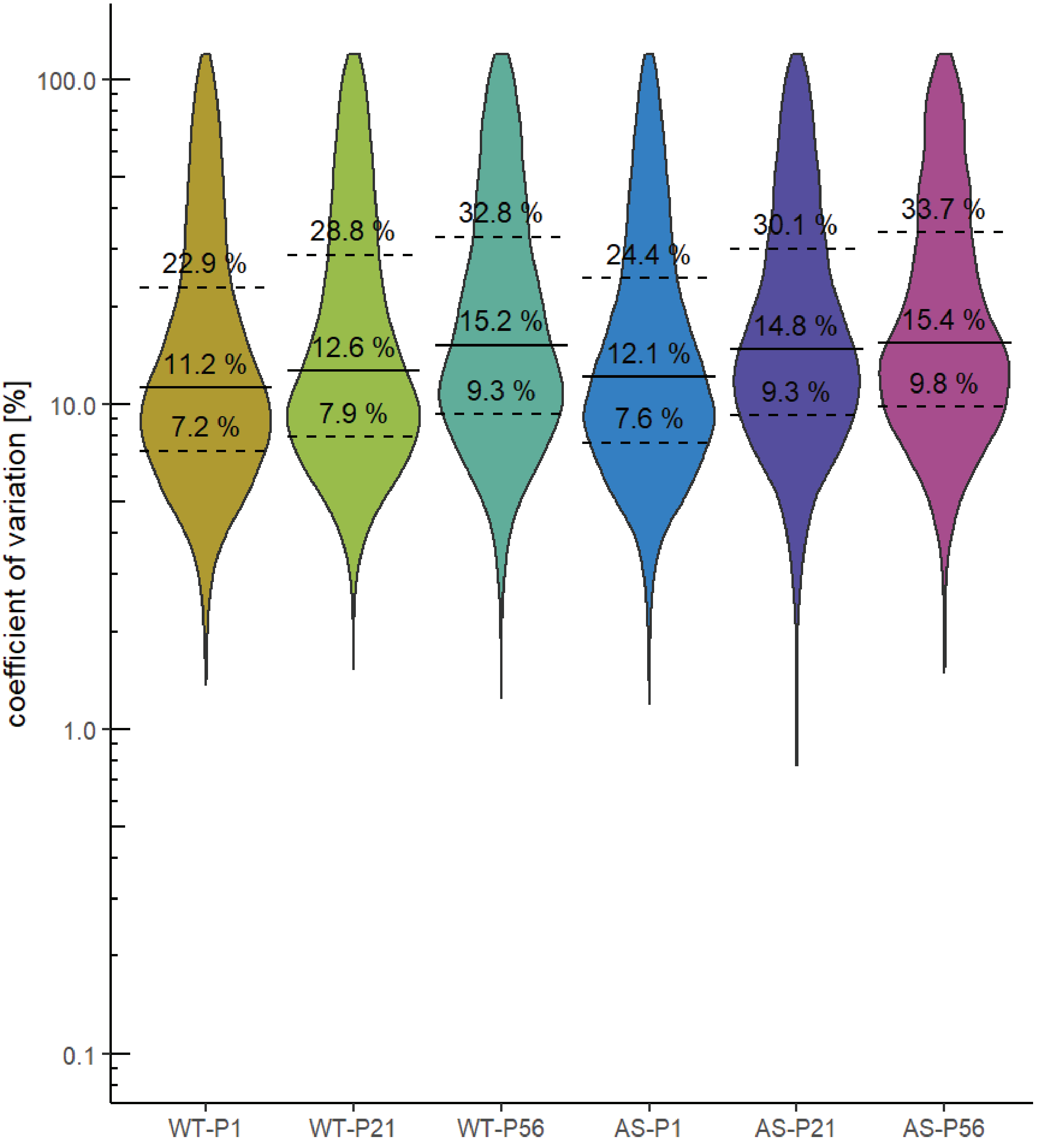

Supplementary Fig. S1: Analytical performance of DIA-MS across mouse cortex samples. Distribution of CV values for the quantified protein groups obtained for DIA as a violin plot. The dark line in the center of each rectangular box is the median of the data, the upper and lower values indicate the 75th and 25th percentiles, and the spikes are the range of the data.. The width of the plot outside the modified box plot is the density of values

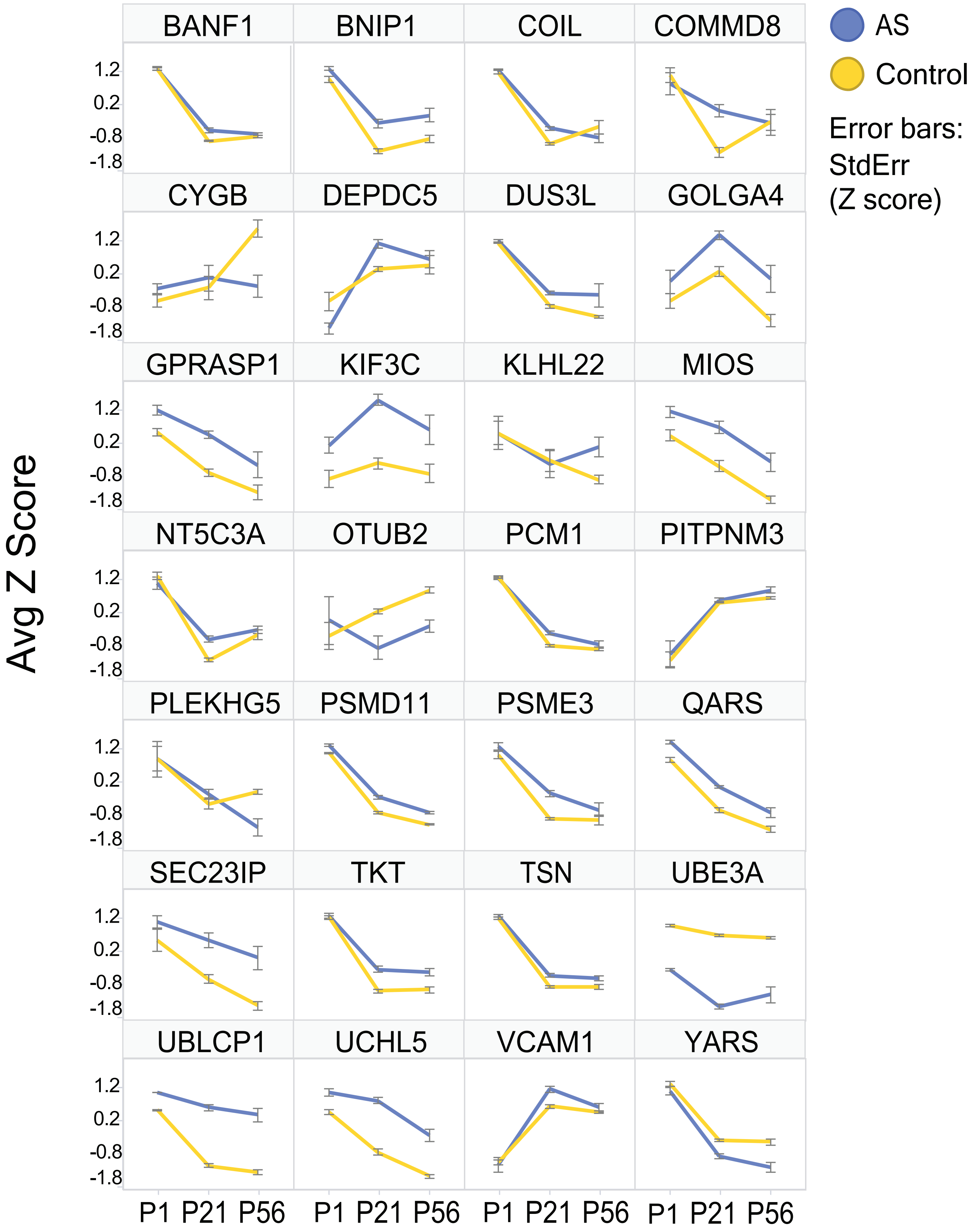

Supplementary Fig. S2: Time course expression of significantly altered (adj. p value < 0.05) proteins in AS vs control mice over the course of cortical development. Values represent Z scored values. Error bars: s.e.m.

A

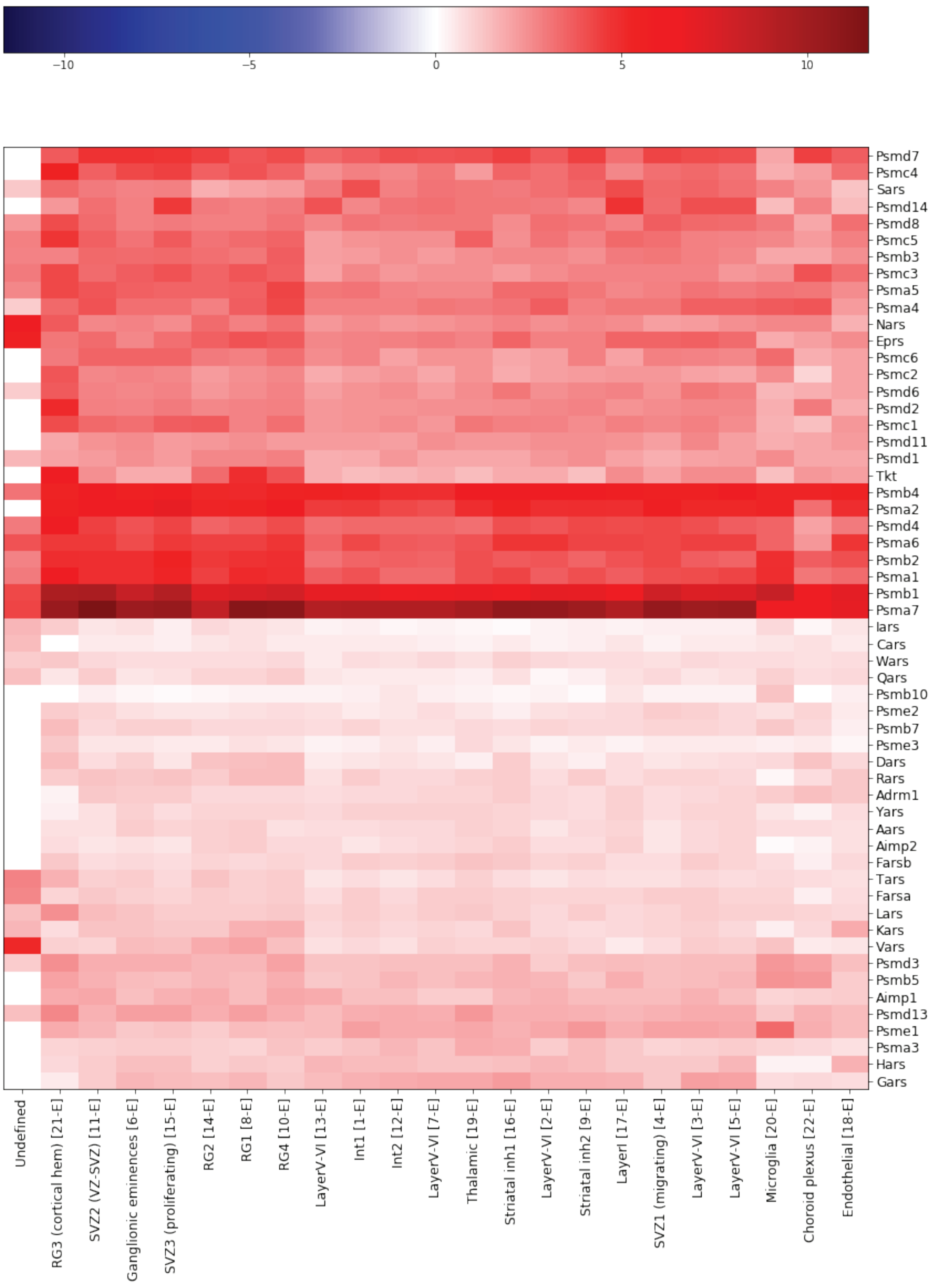
−10
−5
0
5
10
Psmd7
Psmc4
Sars
Psmd14
Psmd8
Psmc5
Psmb3
Psmc3
Psma5
Psma4
Nars
Eprs
Psmc6
Psmc2
Psmd6
Psmd2
Psmc1
Psmd11
Psmd1
Tkt
Psmb4
Psma2
Psmd4
Psma6
Psmb2
Psma1
Psmb1
Psma7
Iars
Cars
Wars
Qars
Psmb10
Psme2
Psmb7
Psme3
Dars
Rars
Adrm1
Yars
Aars
Aimp2
Farsb
Tars
Farsa
Lars
Kars
Vars
Psmd3
Psmb5
Aimp1
Psmd13
Psme1
Psma3
Hars
Gars
Undefined
RG3 (cortical hem) [21-E]
SVZ2 (VZ-SVZ) [11-E]
Ganglionic eminences [6-E]
SVZ3 (proliferating) [15-E]
RG2 [14-E]
RG1 [8-E]
RG4 [10-E]
LayerV-VI [13-E]
Int1 [1-E]
Int2 [12-E]
LayerV-VI [7-E]
Thalamic [19-E]
Striatal inh1 [16-E]
LayerV-VI [2-E]
Striatal inh2 [9-E]
LayerI [17-E]
SVZ1 (migrating) [4-E]
LayerV-VI [3-E]
LayerV-VI [5-E]
Microglia [20-E]
Choroid plexus [22-E]
Endothelial [18-E]

B

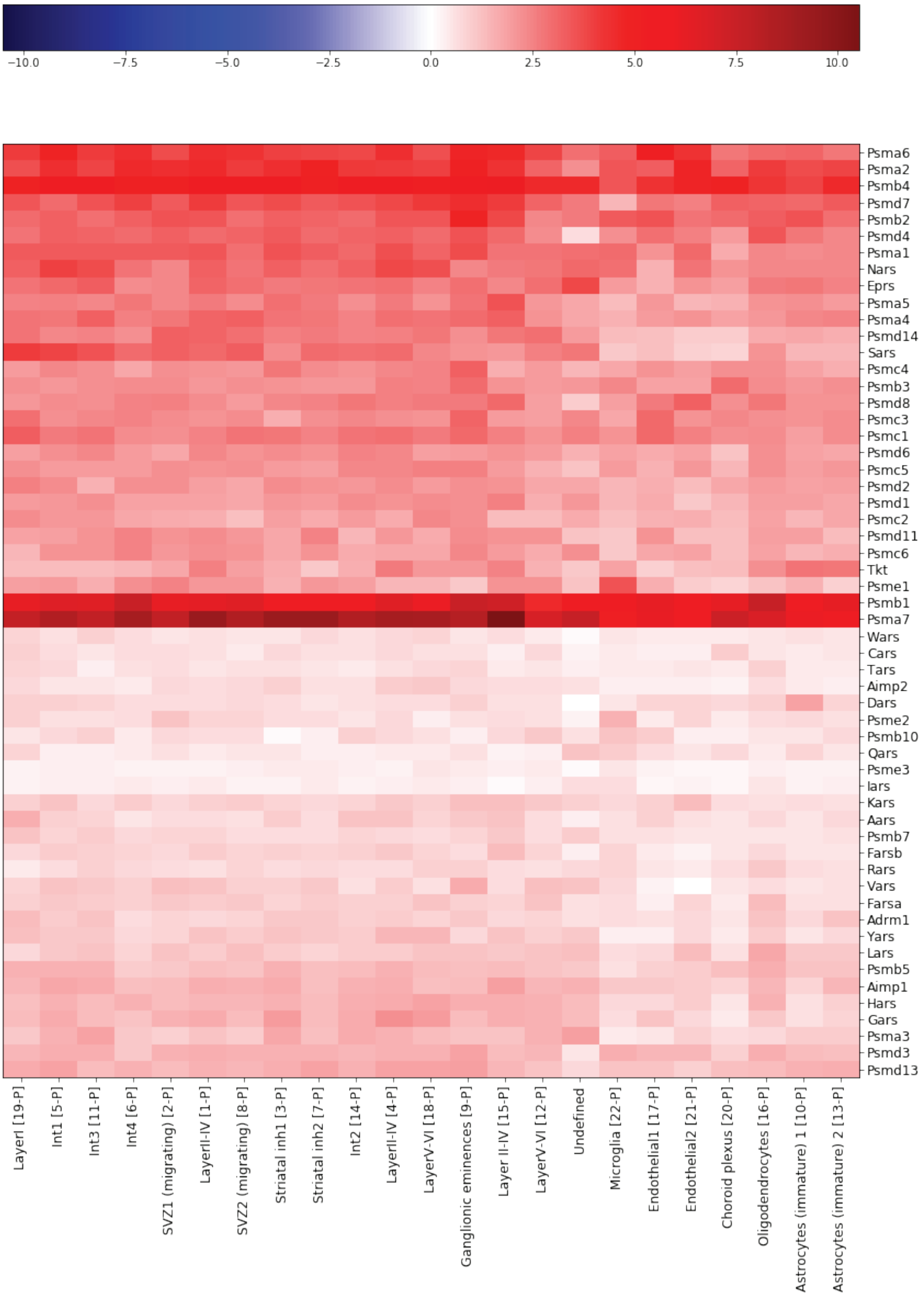

Supplementary Fig. S3: gene expression of top candidates in E14.5 and P0 mouse brains. Gene expression heatmap for pathway components of tRNA synthases, proteasome, and other top candidates, including TKT in A) E14.5 and B) P0 wild type mouse brains. Expression levels are given in transcript counts per 10,000 counts. The data was analyzed from Loo, L., Simon, J.M., Xing, L. et al. Nat Commun 10, 134 (2019).

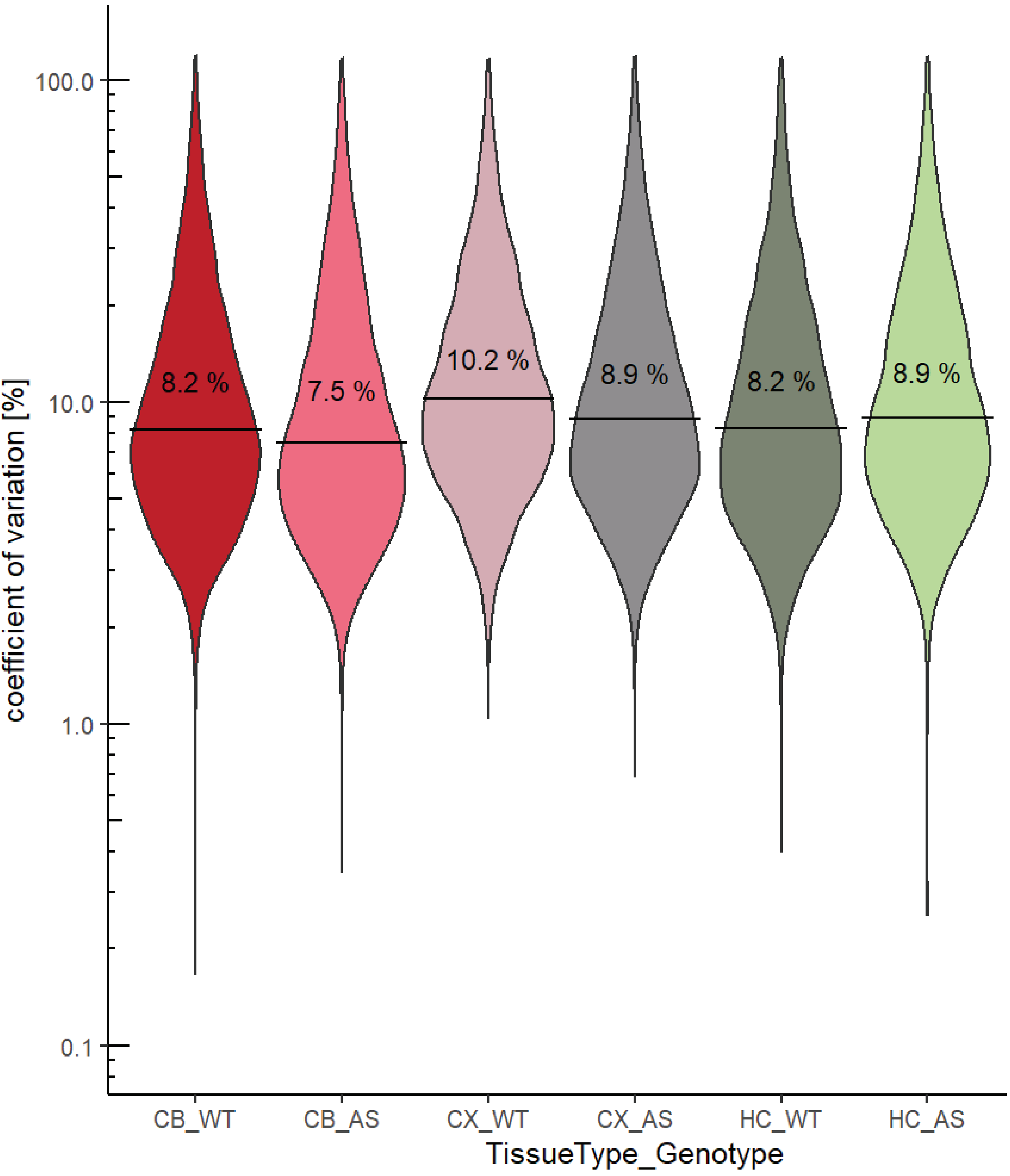

Supplementary Fig. S4: Analytical performance of DIA-MS across rat samples. Distribution of CV values for the quantified protein groups obtained for DIA as a violin plot. The dark line in the center of each rectangular box is the median of the data. The width of the plot outside the modified box plot is the density of values. CB: Cerebellum. CX: Cortex. HC: hippocampus. Genotype: WT: Control. AS: AS.

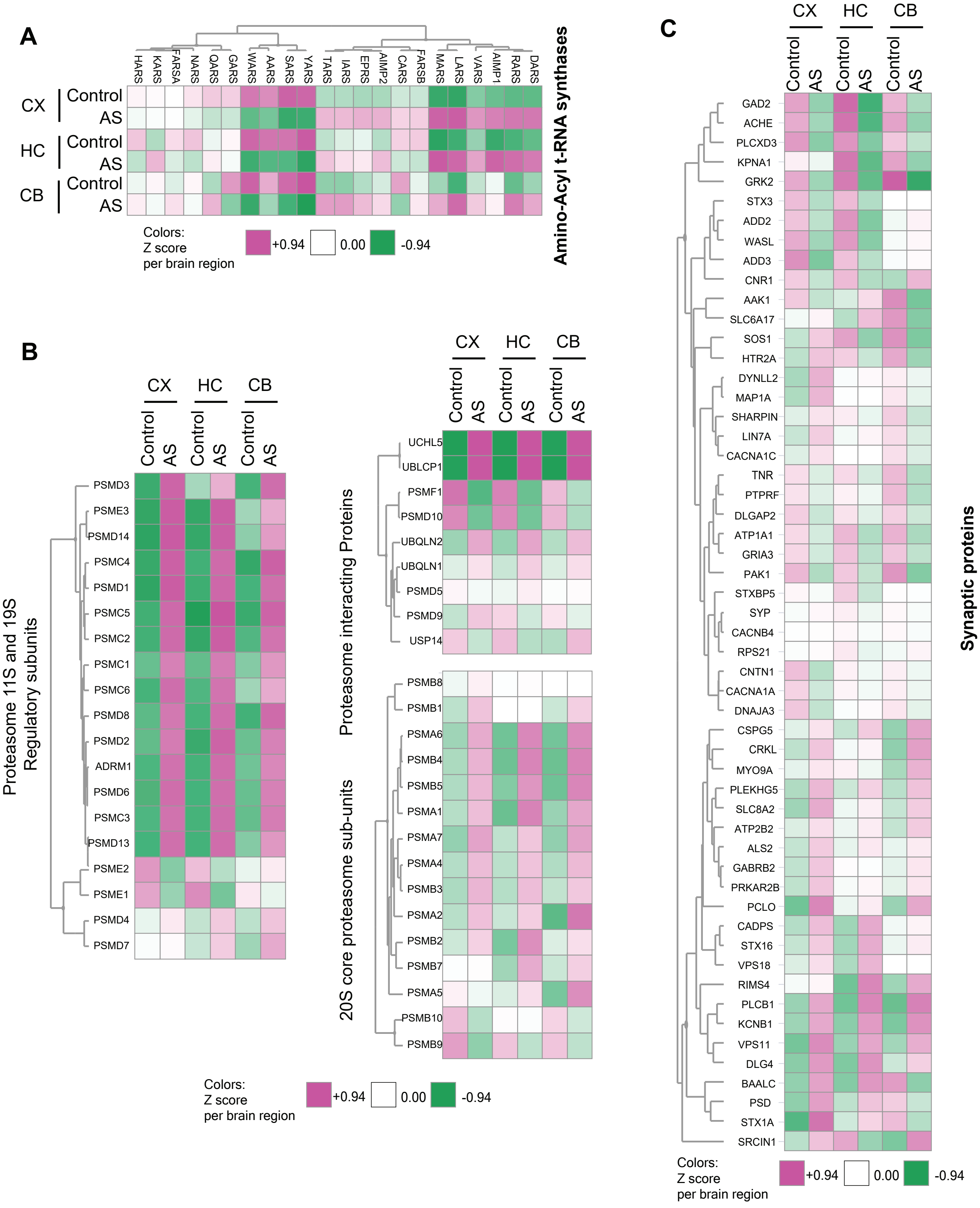

Figure S5: Regulation of amino-acyl t-RNA synthase, proteasome and synaptic proteins in AS rat model.

A) Average Z scored heatmap per time point for amino-acyl tRNA synthases multienzyme complex and amino-acyl tRNA synthatases. Clusters are defined using Euclidian distance based on the UPGMA method.

B) Average Z scored heatmap per time point for proteasome complex based on their sub-unit classification. Clusters are defined using Euclidian distance based on the UPGMA method.

C) Average Z scored heatmap per time point for proteins belonging to the term synapse that were significantly altered (p value < 0.05) at time point P56 in the mouse time course experiment. Clusters are defined using Euclidian distance based on the UPGMA method

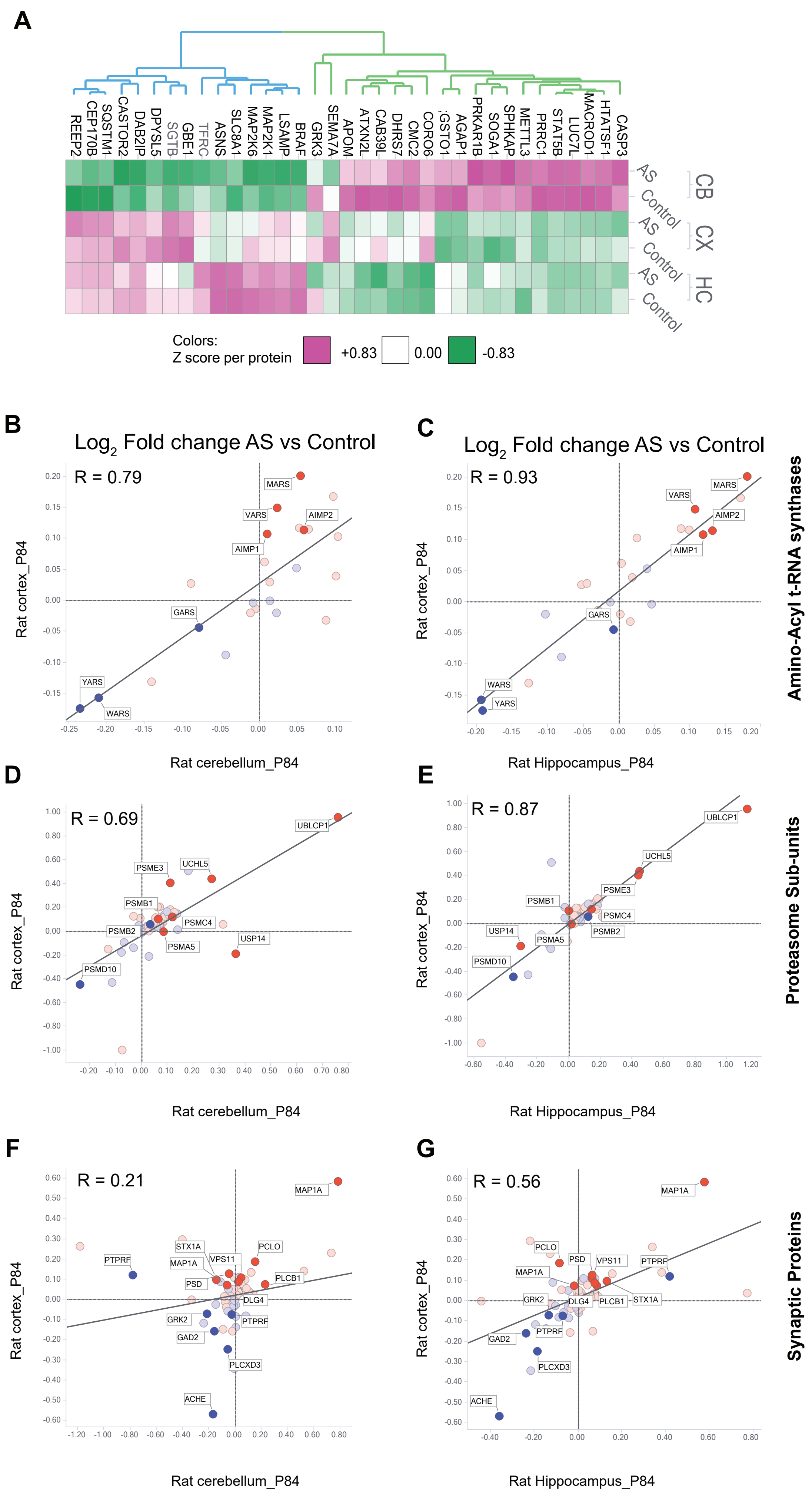
A
REEP2
CEP170B
SQSTM1
CASTOR2
DAB2IP
DPYSL5
SGTB
GBE1
TFRC
ASNS
SLC8A1
MAP2K6
MAP2K1
LSAMP
BRAF
GRK3
SEMA7A
APOM
ATXN2L
CAB39L
DHRS7
CMC2
CORO6
;GSTO1
AGAP1
PRKAR1B
SOGA1
SPHKAP
METTL3
PRRC1
STAT5B
LUC7L
MACROD1
HTATSF1
CASP3
AS
Control
AS
Control
AS
Control
CB
CX
HC
Colors:
Z score per protein
+0.83
0.00
-0.83
B
C
Log2 Fold change AS vs Control
Log2 Fold change AS vs Control
R = 0.79
R = 0.93
Rat cortex_P84
Rat cerebellum_P84
Rat Hippocampus_P84
Amino-Acyl t-RNA synthases
MARS
VARS
AIMP2
AIMP1
GARS
YARS
WARS
D
E
R = 0.69
R = 0.87
UBLCP1
PSME3
UCHL5
PSMB1
PSMB2
PSMC4
PSMA5
USP14
PSMD10
Proteasome Sub-units
F
G
R = 0.21
R = 0.56
MAP1A
PTPRF
STX1A
VPS11
PCLO
PSD
PLCB1
DLG4
GRK2
GAD2
PLCXD3
ACHE
Synaptic Proteins

Supplementary Fig. S6: Analysis of Rat dataset

A) Heatmap of proteins that pass statistical significance (adj. p value < 0.05) in the cerebellum. Proteins fall into two categories. Up regulated in Cerebellum compared to cortex and HC (green cluster) and downregulated in cerebellum compared to cortex (blue cluster).

B -G) Log_2 Fold change co-relation plots between rat cortex and rat cerebellum and rat cortex and rat hippocampus for proteins in the amino-acyl t-RNA synthases pathway (B,C), Proteasome sub-units (D,E) and synaptic proteins (F,G) as filtered in figure 2F. Correlation coefficients are calculated using Pearson's method.

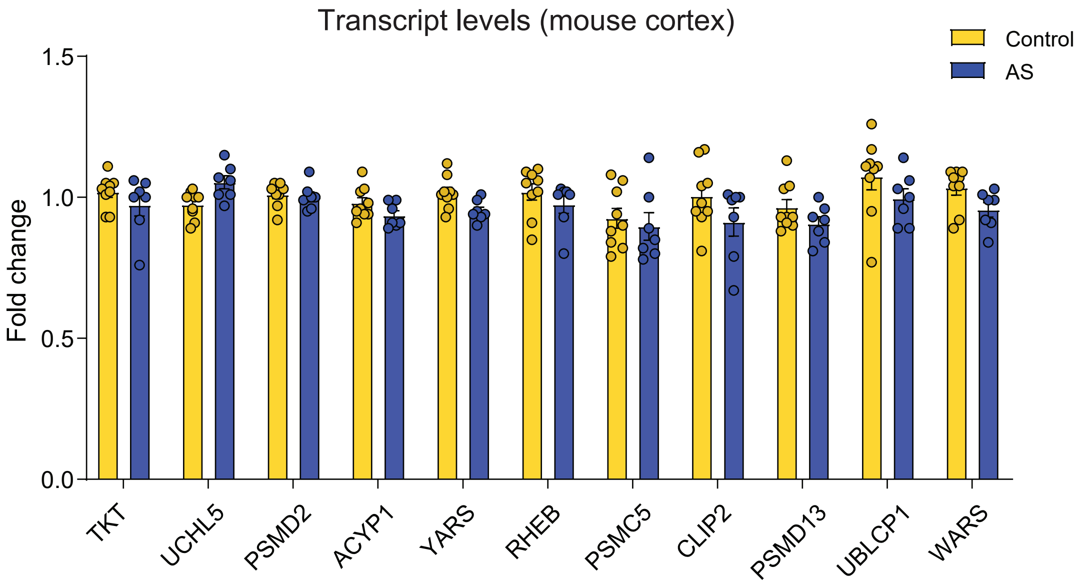

Supplementary Fig. S7: Transcript levels of putative UBE3A targets are unchanged in cortical samples of AS mice

qRT-PCR analysis of messenger RNA of top altered proteins in AS mice compared to control. Changes in transcript levels between control and AS are non-significant. Statistical testing was performed using multiple t tests.

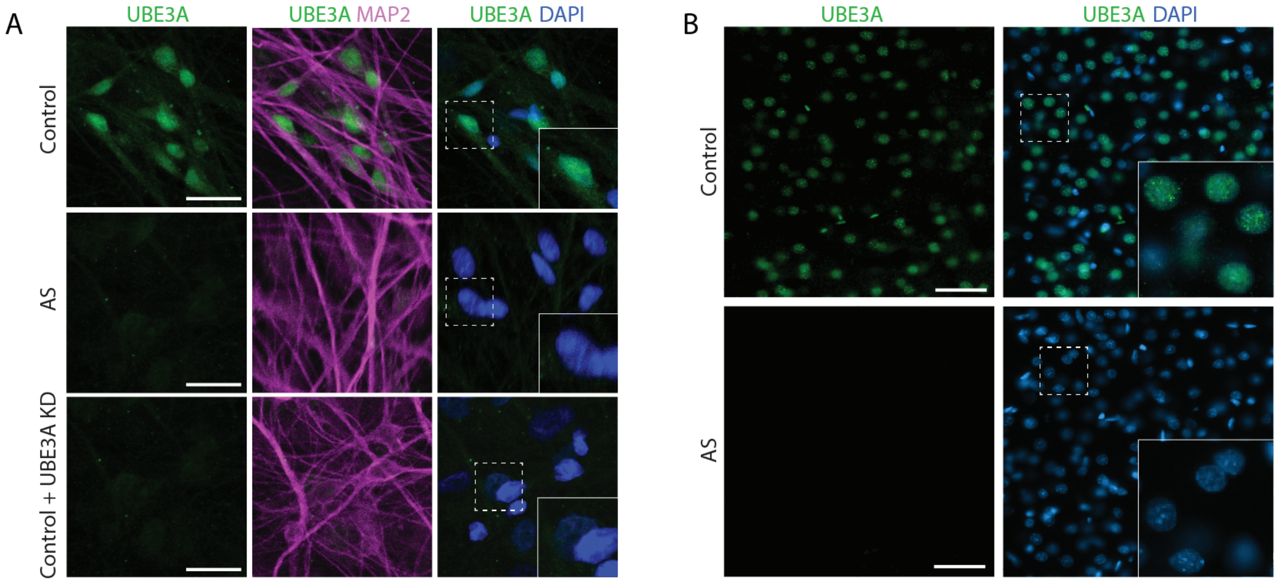

Supplementary Fig. S8: UBE3A expression is undetectable AS hiPSC neurons and AS rat visual cortex

A) Immunocytochemical images of UBE3A and the neuronal marker MAP2 in hiPSC-derived neurons of control, control + UBE3A KD ASO, and AS lines. Nuclei were counterstained with DAPI. Scale bars: 25 µm.
B) Immunohistochemical images of UBE3A and the neuronal marker NEUN in the primary visual cortex of adult control and AS rats. Nuclei were counterstained with DAPI. Scale bars: 50 µm.

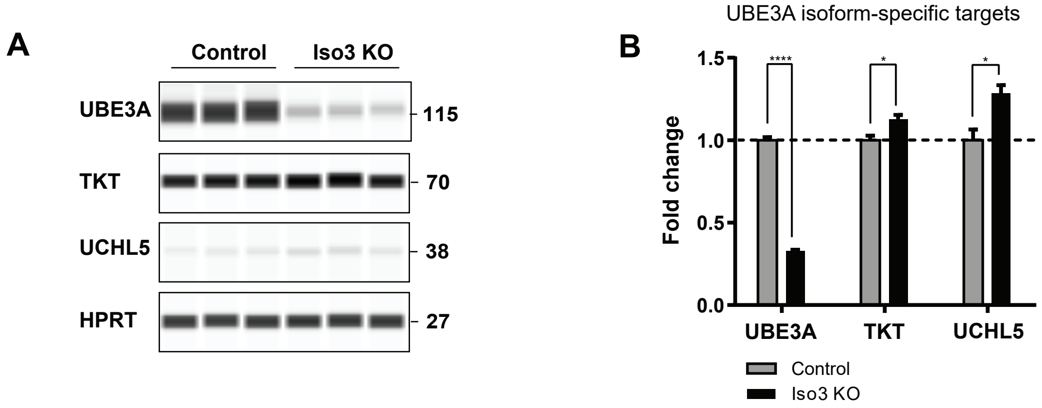

Supplementary Fig. S9: Capillary western blot analysis of TKT and UCHL5 in mice lacking the nuclear (Iso3 KO) UBE3A.

A) Digital rendering of capillary electrophoresis bands control and Iso3 KO mice
B) Quantification of relative protein abundance measured with capillary electrophoresis (* $p < 0.05$, **** $p < 0.0001$).

A

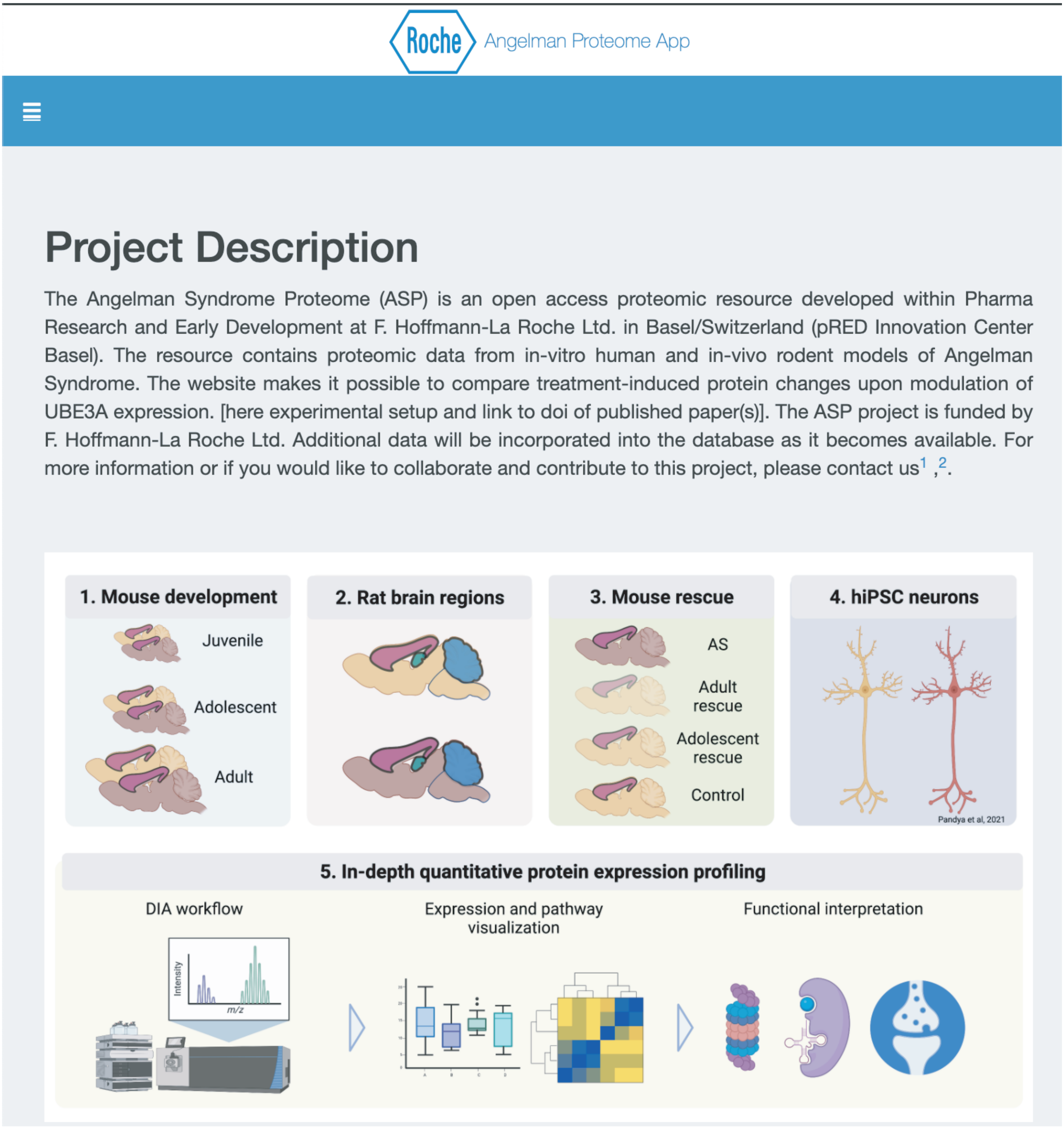

Supplementary Fig. S10: angelman-proteome-project.org wesbite.
A) Welcome page of the Angelman proteome project website.
B) Example box plot for UBE3A expression in human iPSC control and AS neurons, mouse developmental time points, UBE3A expression upon UBE3A rescue at P21 and P56 and AS rat brain in Cerebellum (CB), Cortex (CX) and Hippocampus (HC).
C) Example box plot for TKT expression as in B).
D) Example pathway visualization of the proteasome pathway in the UBE3A rescue dataset.

B

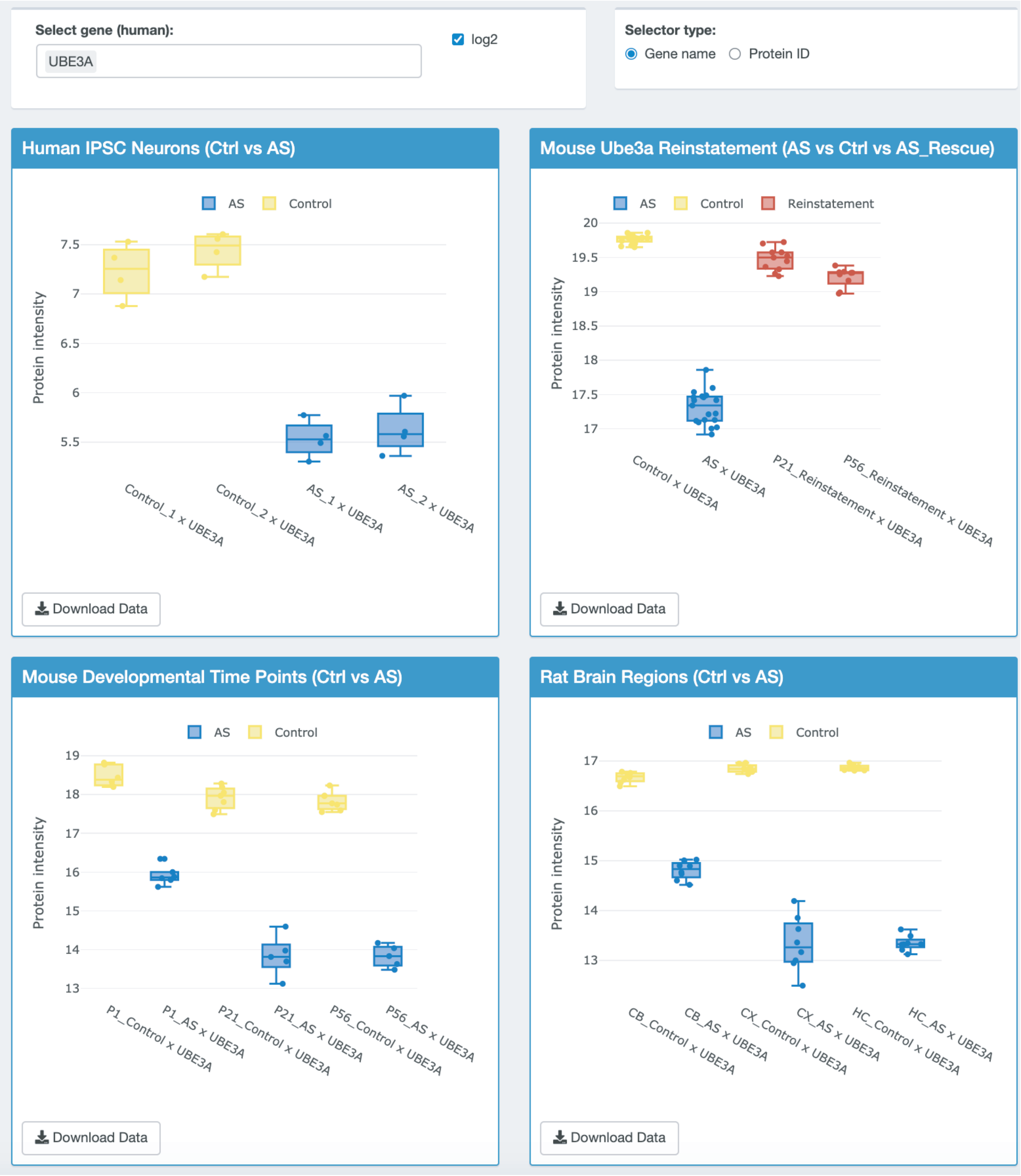

Select gene (human):
UBE3A
log2
Selector type:
Gene name
Protein ID
Human IPSC Neurons (Ctrl vs AS)
AS
Control
Protein intensity
7.5
7
6.5
6
5.5
Control_1 x UBE3A
Control_2 x UBE3A
AS_1 x UBE3A
AS_2 x UBE3A
Download Data
Mouse Ube3a Reinstatement (AS vs Ctrl vs AS_Rescue)
AS
Control
Reinstatement
Protein intensity
20
19.5
19
18.5
18
17.5
17
Control x UBE3A
AS x UBE3A
P21_Reinstatement x UBE3A
P56_Reinstatement x UBE3A
Download Data
Mouse Developmental Time Points (Ctrl vs AS)
AS
Control
Protein intensity
19
18
17
16
15
14
13
P1_Control x UBE3A
P1_AS x UBE3A
P21_Control x UBE3A
P21_AS x UBE3A
P56_Control x UBE3A
P56_AS x UBE3A
Download Data
Rat Brain Regions (Ctrl vs AS)
AS
Control
Protein intensity
17
16
15
14
13
CB_Control x UBE3A
CB_AS x UBE3A
CX_Control x UBE3A
CX_AS x UBE3A
HC_Control x UBE3A
HC_AS x UBE3A
Download Data

C

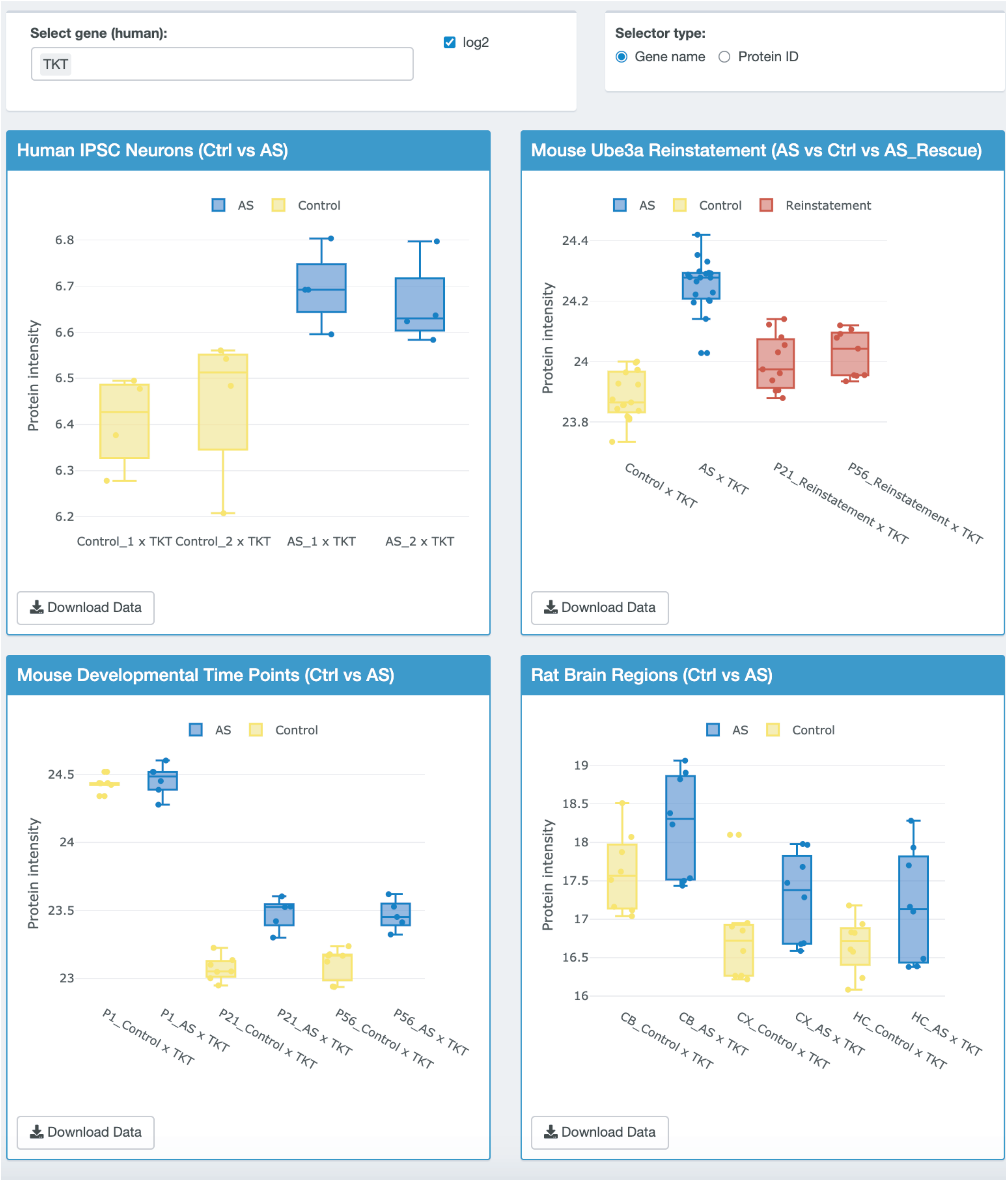

Select gene (human):
TKT
log2
Selector type:
Gene name
Protein ID
Human IPSC Neurons (Ctrl vs AS)
AS
Control
Protein intensity
6.8
6.7
6.6
6.5
6.4
6.3
6.2
Control_1 x TKT
Control_2 x TKT
AS_1 x TKT
AS_2 x TKT
Download Data
Mouse Ube3a Reinstatement (AS vs Ctrl vs AS_Rescue)
AS
Control
Reinstatement
Protein intensity
24.4
24.2
24
23.8
Control x TKT
AS x TKT
P21_Reinstatement x TKT
P56_Reinstatement x TKT
Download Data
Mouse Developmental Time Points (Ctrl vs AS)
AS
Control
Protein intensity
24.5
24
23.5
23
P1_Control x TKT
P1_AS x TKT
P21_Control x TKT
P21_AS x TKT
P56_Control x TKT
P56_AS x TKT
Download Data
Rat Brain Regions (Ctrl vs AS)
AS
Control
Protein intensity
19
18.5
18
17.5
17
16.5
16
CB_Control x TKT
CB_AS x TKT
CX_Control x TKT
CX_AS x TKT
HC_Control x TKT
HC_AS x TKT
Download Data

D

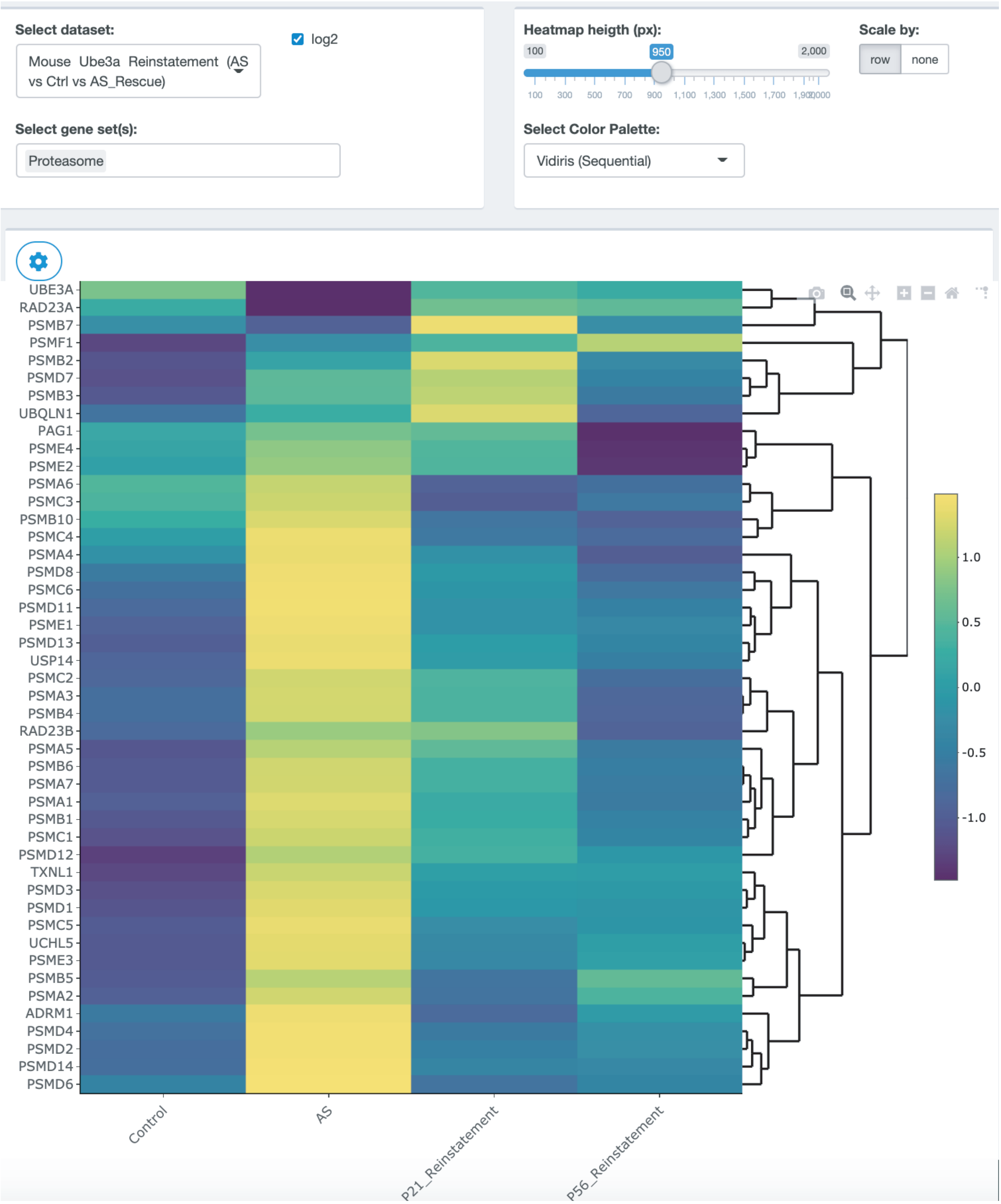

Select dataset:
Mouse Ube3a Reinstatement (AS vs Ctrl vs AS_Rescue)
log2
Select gene set(s):
Proteasome
Heatmap heigth (px):
100
950
2,000
100 300 500 700 900 1,100 1,300 1,500 1,700 1,900 2,000
Scale by:
row
none
Select Color Palette:
Vidiris (Sequential)
UBE3A
RAD23A
PSMB7
PSMF1
PSMB2
PSMD7
PSMB3
UBQLN1
PAG1
PSME4
PSME2
PSMA6
PSMC3
PSMB10
PSMC4
PSMA4
PSMD8
PSMC6
PSMD11
PSME1
PSMD13
USP14
PSMC2
PSMA3
PSMB4
RAD23B
PSMA5
PSMB6
PSMA7
PSMA1
PSMB1
PSMC1
PSMD12
TXNL1
PSMD3
PSMD1
PSMC5
UCHL5
PSME3
PSMB5
PSMA2
ADRM1
PSMD4
PSMD2
PSMD14
PSMD6
Control
AS
P21_Reinstatement
P56_Reinstatement
1.0
0.5
0.0
-0.5
-1.0
