# Supplementary material for: A cross-species spatiotemporal proteomic analysis identifies UBE3A-dependent signaling pathways and targets
Source: Mol Psychiatry. 2022 Mar 9;27(5):2590–601. doi: 10.1038/s41380-022-01484-z (PMC9135630; doi:10.1038/s41380-022-01484-z)
Supplement: Supplementary file 11 — Supplementary Methods [file 41380_2022_1484_MOESM11_ESM.docx]

## **Supplementary Methods: Pandya et al**

### Animals

Animals were age and gender matched and a combination of male and female mice were used for the experiments. Mice were genotyped when they were 7-10 days, and re-genotyped at the moment the mice were sacrificed. Genotyping records were obtained and kept by a technician not involved in the experimental design, performance and analysis. Cages were semi-randomly (alternatingly) assigned to either treat all mice within the cage with vehicle or with tamoxifen and genotypes. Both male and female mice were used. The alternating randomization was adjusted if there was an imbalance of genotype or sex.

### Sample preparation

Tissue samples were denatured using Biognosys’ Denature Buffer, and reduced and alkylated using Biognosys’ Reduction and Alkylation Solution for 60 min at 37°C. Subsequently, digestion to peptides was carried out using trypsin (w/w ratio 1 50 Promega) overnight at 37°C. Samples were prepared using the PreOmics sample preparation kit and frozen as dried peptides. Peptides were resuspended in LC solvent A (1 % acetonitrile, 0.1 % formicacid (FA)) spiked with Biognosys’ iRT kit calibration peptides. Peptide concentrations were determined using a UV/VIS Spectrometer (SPECTROstar Nano, BMG Labtech).

### HPRP fractionation

Cortex samples (P1, P21, P56): Peptides were pooled according to the genotype (two pools: WT and AS). Ammonium hydroxide was added to both pools to a pH value > 10. The fractionation was performed using a Dionex UltiMate 3000RS pump (Thermo Scientific) on an Acquity UPLC CSH C18 1.7 µm, 2.1 x 150 mm column (Waters). The gradient was 1 % to 40 % solvent B in 20 minutes, solvents were A: 20 mM ammonium formate in water, B: Acetonitrile. Fractions were taken every 30 seconds and sequentially pooled to 6 fraction pools for mouse cortex samples and 8 for rat brain samples. For mouse cortex samples, the eluates were dried down, resolved in 15 µl solvent A, and spiked with Biognosys’ HRM kit calibration peptides, rat samples were resolved in 12 µL solvent A and spiked with Biognosys’ iRT kit calibration peptides.

Mouse cortex samples (Ube3a reinstatement): Two pools of peptides were generated (WT; CreERT+ and Ube3aStop/p+; CreERT-, 6 samples each). The two pools were diluted 4x in 0.2 M ammonium formate (pH 10) and applied on C18 MicroSpin columns (The Nest Group). The peptides were then eluted with buffers containing 0.05 M ammonium formate and increasing acetonitrile concentrations (5 %, 10 %, 15 %, 20 %, 25 %, and 50 %) at a pH of 10. The eluates were dried down, resolved in 15 µl solvent A and spiked with Biognosys’ HRM kit calibration peptides. Fractions 5 % and 50 % were pooled.

All peptide concentrations were determined using a UV/VIS Spectrometer.

### Shotgun LC MS/MS for spectral library generation

For the LC MS/MS measurements of mouse brain tissues, 2 µg of peptides per fraction (or 4 µg for fraction 5+50 % of cortex samples with Ube3a reinstatement) were injected to an in house packed C18 column (Dr. Maisch ReproSil Pur 1.9 µm particle size, 120 Å pore size 75 µm inner diameter, 50 cm length, New Objective) on a Thermo Scientific™ Easy nLC 1200 nano liquid chromatography system connected to a Thermo Scientific Q Exactive™ HF mass spectrometer equipped with a standard nano electrospray source LC solvents were A: 1 % acetonitrile in water with 0.1 % FA; B: 15 % water in acetonitrile with 0.1 % FA. The LC gradient was 1-55 % solvent B in 60 minutes followed by 55-90 % B in 10 seconds, 90 % B for 10 minutes, 90 % - 1 % B in 0.1 minutes and 1 % B for 5 minutes. A modified TOP15 methods was used [1]

Additionally, mouse Somatosensory cortex 1 barrel field and mouse cerebellum libraries from MCP publication by Bruderer *et al*. [2] were used for the analysis.

For DDA LC MS/MS measurements of rat brain samples, 1 µg of peptides per fraction were injected to an in-house packed reversed phase column (PicoFrit emitter with 75 µm inner diameter, 60 cm length and 10 µm tip from New Objective, packed with 1.7 µm Charged Surface Hybrid C18 particles from Waters) on a Thermo Scientific EASY-nLC ™ 1200 nano-liquid chromatography system connected to a Thermo Scientific Orbitrap Fusion Tribrid mass spectrometer equipped with a Nanospray Flex™ Ion Source. LC solvents were A: 1 % acetonitrile in water with 0.1 % FA; B: 20 % water in acetonitrile with 0.1 % FA. The nonlinear LC gradient was 1 – 59 % solvent B in 95 min followed by 59 – 90 % B in 10 seconds, 90 % B for 8 min, 90 % - 1 % B in 10 seconds and 1 % B for 5 min at 60°C and a flow rate of 250 nl/min. A modified top speed method (3 s cycle time) from Hebert et al. was used [3].

### Database search of shotgun LC MS/MS data

For developmental time course cortex samples (P1, P21, P56), the mass spectrometric data were analyzed using Biognosys’ search engine SpectroMine™, and for cortex samples with Ube3a reinstatement, Biognosys’ search engine Pulsar (version 1.0.19846) was used. The false discovery rate on peptide and protein level was set to 1 %. A mouse UniProt fasta database (Mus musculus, 2019-07-01) was used for the search engine, allowing for 2 missed cleavages and variable modifications (N-term acetylation, methionine oxidation).

For rat brain tissue samples, mass spectrometric data were analyzed using Biognosys’ search engine SpectroMine™ (version 1.0.190808), the false discovery rate on peptide and protein level was set to 1 %. A rat UniProt/Trembl. Fasta database (Rattus norvegicus, 2019-07-01) was used for the search engine, allowing for 2 missed cleavages and variable modifications (N-term acetylation, methionine oxidation).

### HRM ID+ mass spectrometry acquisition

For the LC MS/MS HRM measurements, 2 µg of peptides per sample were injected to an in house packed C18 column (Dr. Maisch ReproSil Pur, 1.9 µm particle size, 120 Å pore size; 75 µm inner diameter, 50 cm length, New Objective) on a Thermo Scientific Easy nLC 1200 nano liquid chromatography system connected to a Thermo Scientific Q Exactive HF mass spectrometer equipped with a standard nano electrospray source. LC solvents were A: 1 % acetonitrile in water with 0.1 % FA; B: 15 % water in acetonitrile with 0.1 % FA. The nonlinear LC gradient was 1-55 % solvent B in 120 minutes followed by 55-90 % B in 10 seconds and 90 % B for 10 minutes.

## **HRM data analysis**

### Mouse developmental time course

N = 6 (P1 WT and AS), 7 (P21 WT), 5 (P21 AS), 7 (P56 WT) and 5 (P56 AS) were used in the study. No prior sample size estimation was used for this discovery experiment. Statistical and Bioinformatics analysis was performed in the R statistical environment [4] and the Perseus software [5].

Protein intensities <20 on the original scale were considered to be below the noise threshold and were marked as missing. Data were log2-transformed and filtered to contain 50% valid values across all samples. Due to the completeness of DIA data, only 3 proteins were excluded and a total of 7,184 protein groups were retained for further analysis (Supplementary Table 1).

Partial Least Squares Discriminant Analysis (PLS-DA) was conducted using the DiscriMiner R Package [6] with default settings and was based on the completely measured 7,029 protein groups.

To identify differentially expressed proteins we employed the samr R package [7], which allows for permutation-based false discovery rate control and reports the associated q-value [8]. The Two-Class Unpaired Test with 100 permutations was used to compare AS and WT genotypes at each time point separately. Proteins were classified as statistically significantly differentially expressed, if they met a q-value threshold of 5% (Supplementary Table 2).

Pathway analysis was conducted in the Perseus software using the 1D annotation function. Gene Ontology (GO) annotations for each protein were downloaded from UniProt [9]. As multiple enrichment tests were performed, the Benjamini-Hochberg method was used to correct for multiple-hypothesis testing. For completeness pathway enrichment scores are plotted for all 3 comparisons and the significantly regulated ones are marked explicitly (Supplementary Table 3).

**Rat brain regions**

N = 8 WT and AS rats were used per brain region. A similar analysis strategy as described above was applied to the rat data set. Briefly, protein expression values below 20 were converted to NAs. Data were log2-transformed and filtered to retain protein groups for which values were measured in at least 50% of all samples, resulting in 7,524 protein groups (Supplementary Table 4). PLS-DA analysis was conducted using proteins with measurements across all samples (7,346) using the DiscriMiner R package [6]. Differentially expressed proteins were identified between KO and WT rat samples across each brain region separately. The samr function for Two Class Unpaired Test was used and q-value of 0.05 was considered to mark proteins as statistically significantly differentially expressed (Supplementary Table 5).

### Mouse Ube3a reinstatement

N = 9 WT and AS rats were used per brain region Protein expression values below 20 were considered below the detection limit and converted to NAs and data were log2-transformed. 5,325 protein groups contained measured values in at least 50% of all samples and were used for the subsequent statistical analysis (Supplementary Table 6). PLS-DA analysis was conducted on proteins measured across all samples (5,314) using the DiscriMiner R package [6].

The samr function with response type set to ‘multiclass’ from the package was used to identify differentially expressed protein groups across 4 conditions: WT, KO, Rescue at p21 and Rescue at p56. To determine which pairwise comparisons were significant the Tukey’s Honestly Significant Difference test from the R stats package [7] was used and the alpha level was set to 5% (Supplementary Table 7).

To identify up- or down-regulated pathways, the 1D annotation test in the Perseus software was used. For each pairwise comparison KO vs. WT, Rescue p21 vs. KO, and Rescue p56 vs. KO, proteins were first mapped to GO annotations and keywords downloaded from the Uniprot database. Subsequently, enrichment scores were calculated and multiple hypothesis testing correction using the Benjamini-Hochberg procedure applied to identify statistically significant differences. For completeness, pathway enrichment scores were always plotted for all 3 comparisons and the significantly regulated ones are marked explicitly (Supplementary Table 8).

This paper does not report original code.

### Species overlap

To investigate the overlap in UBE3A targets across the three species, we compared proteins with (1) increased expression in AS individuals compared to Controls (fold change > 0.25 log2) and (2) with decreased expression in AS individuals compared to Controls (fold change < -0.25) across the species. Proteins across the three species were mapped using the InParanoid database for orthologs groups. The RVenn package was used to visualize the numbers of overlapping proteins across all comparisons between the species.

### Capillary western blot

Briefly, after protein extraction and quantification, a final sample concentration of 0.25 mg/ml was loaded to the capillary cartridges (12–230 kDa Peggy Sue or Sally Sue Separation Module, #SM-S001). Chemiluminescent protein detection was performed using the Anti Rabbit and Anti Mouse Detection Modules (#DM-001 and #DM-002). The analysis of relative protein expression was carried out with the Compass for SW software (Version 4.1.0, Protein Simple) and statistical analysis with GraphPad Prism Software (Version 8) using ANOVA followed by Tukey’s post hoc test. Each experiment was performed using 3 independent biological replicates of hiPSC neurons (Fig. 4F, 5A) and 3 mice (Supplementary Fig. S9).

### Antibody panel

| Antigen | Company | Cat. No. | Dilution mouse brain lysates | Dilution hiPSC lysates |
| --- | --- | --- | --- | --- |
| Actin |  |  | N/A | 1:2,000 |
| Acyp1 | Abcam | ab2313231 | 1:200 | 1:50 |
| Clip2 | Abcam | ab224111 | 1:800 | 1:2,000 |
| Dzank1 | Abcam | ab221461 | 1:25 | N/A |
| Psmd2 | Abcam | ab140675 | 1:800 | 1:200 |
| Psme3 | Abcam | ab157157 | 1:500 | 1:100 |
| Rheb | Abcam | ab25873 | 1:250 | 1:50 |
| Sod2 | Abcam | ab13533 | 1:1000 | 1:20,000 |
| Tkt | Sigma-Aldrich | HPA029480 | 1:50 | 1:1,000 |
| Ube3a | Bethyl | A300-352A | 1:100 | 1:200 |
| Ublcp1 | Abcam | ab176340 | N/A | 1:500 |
| Ublcp1 | LSBio | LS-C157967 | 1:2,000 | N/A |
| Uchl5 (Uch37) | Bethyl | A304-099A | 1:1,000 | 1:1,000 |
| Usp14 | Abcam | ab137432 | 1:500 | 1:1,000 |
| Vinculin | Abcam | ab129002 | 1:200 | N/A |
| Wars | Bethyl | A304-274A | N/A | 1:10,000 |
| Yars | Thermo Fisher | PA5-53883 | 1:100 | 1:200 |

### hIPSC culture

All lines were tested for mycoplasma contamination at various states of the project from hiPSCs to NPCs to neurons. Research performed on samples of human origin was conducted following informed consent, as approved by the institutional review board of Boston Children’s Hospital. NPCs between passage numbers 15–25 were used for the entire analysis. Neuronal cell culture media: N2B27 medium is a 1:1 mixture of DMEM/F12 (1:1) medium with GlutaMAX I and Neurobasal medium, containing 1 × N2 supplement, 1 × B27 supplement minus Vitamin A, and 50 μM βmercaptoethanol (all from LifeTechnologies). NEP medium is N2B27 medium containing 5 ng/ml FGF-2, 250 ng/ml noggin (R&D Systems or Peprotech), and 20 μM SB 431542 (Tocris). FEB medium is N2B27 medium containing 10 ng/ml FGF-2 (Peprotech), 10 ng/ml EGF (R&D Technologies), and 20 ng/ml BDNF (Peprotech). SFA medium is N2B27 medium containing 100 ng/ml FGF-8 (Peprotech), 200 ng/ml sonic hedgehog (Peprotech) and 100 μM ascorbic acid 2-phosphate (Sigma). BGAA medium is N2B27 medium containing 20 ng/ml BDNF, 10 ng/ml GDNF (Peprotech), 500 μM dibutyryl cyclic AMP (Sigma) and 100 μM ascorbic acid 2- phosphate. Generation of neural precursor cells (NPCs). NPCs were generated from hESCs using a modified dual SMAD inhibition protocol. hiPSCs were dissociated to single cells and plated into AggreWell800 plates (STEMCELL Technologies) at a density of 5,000 cells per microwell in NEP medium supplemented with 10 μM Y-27632. After five days, aggregates were recovered, plated on polyornithine-/laminin-coated (PL) dishes in NEP medium and cultured for an additional three days to form neural rosettes. Rosettes were isolated manually and replated on PL dishes in NEP medium. Upon reaching confluence, cells were dissociated with 0.05% trypsin/EDTA solution (LifeTechnologies) and plated at 100,000 cells/cm2 on PL dishes in FEB medium. Cells were cultured under these conditions with passaging every 2 – 3 days for ca. 15 population doublings (PDs), followed by a stepwise decrease in plating density to 25,000 cells/cm2 within ca. 10 PDs. Medium was replaced daily throughout the entire NPC derivation procedure. NPC lines were characterized by FACS and immunocytochemical analyses. Neuronal differentiation. NPCs were dissociated with trypsin/EDTA, plated on PL dishes at 10,000 – 15,000 cells/cm2 in SFA medium, and cultured for one week with medium replacement after four days. The resultant progenitors were dissociated with trypsin/EDTA, plated on PL dishes at 35,000 – 50,000 cells/cm2 in BGAA medium and differentiated for up to 6 weeks with biweekly medium replacement.

### ASO treatments

| ASO name | Target | Sequence : Characters in bold are nucleotides that contain a C2'-endo (alpha-L-LNA) conformation. |
| --- | --- | --- |
| UBE3A KD | UBE3A mRNA | TTTAcacctacttcttaaCA |
| NT | NA | TTGaataagtggaTGT |

### Immunohistochemistry

Briefly, sections were blocked with PBS containing 0.5% Triton X-100 and 5% normal horse serum for 1 h at room temperature. Primary antibody labeling was performed in PBS containing 0.5% Triton X-100 and 1% normal horse serum overnight at room temperature. Following primary antibody labeling, sections were washed with PBS and incubated with corresponding Alexa-conjugated secondary antibodies and DAPI in PBS buffer containing 0.5% Triton X-100, 1% normal horse serum for 2 h at room temperature.

**Supplementary Method References**

1. Scheltema RA, Hauschild J-P, Lange O, Hornburg D, Denisov E, Damoc E, et al. The Q Exactive HF, a Benchtop Mass Spectrometer with a Pre-filter, High-performance Quadrupole and an Ultra-high-field Orbitrap Analyzer. Mol Cell Proteomics. 2014;13:3698–3708.

2. Bruderer R, Bernhardt OM, Gandhi T, Xuan Y, Sondermann J, Schmidt M. Optimization of Experimental Parameters in Data-Independent Mass Spectrometry Significantly Increases Depth and Reproducibility of Results. Mol Cell Proteomics. 2017;41.

3. Hebert AS, Richards AL, Bailey DJ, Ulbrich A, Coughlin EE, Westphall MS, et al. The one hour yeast proteome. Mol Cell Proteomics. 2014. 2014. https://doi.org/10.1074/mcp.M113.034769.

4. Team RC. R: A Language and Environment for Statistical Computing. Vienna, Austria. 2019. 2019.

5. Tyanova S, Temu T, Sinitcyn P, Carlson A, Hein MY, Geiger T, et al. The Perseus computational platform for comprehensive analysis of (prote)omics data. Nat Methods. 2016.

6. Gaston A, Sanchez MG. DiscriMiner : Tools of the Trade for Discriminant Analysis. R Packag Version 01-29. 2013. 2013.

7. Rédei GP. SAM (significance analysis of microarrays). Encycl. Genet. Genomics, Proteomics Informatics, 2008.

8. Storey JD. A direct approach to false discovery rates. J R Stat Soc Ser B Stat Methodol. 2002. 2002. https://doi.org/10.1111/1467-9868.00346.

9. Bateman A. UniProt: A worldwide hub of protein knowledge. Nucleic Acids Res. 2019. 2019. https://doi.org/10.1093/nar/gky1049.
