# Supplementary material for: A cross-species spatiotemporal proteomic analysis identifies UBE3A-dependent signaling pathways and targets
Source: Mol Psychiatry. 2022 Mar 9;27(5):2590–601. doi: 10.1038/s41380-022-01484-z (PMC9135630; doi:10.1038/s41380-022-01484-z)
Supplement: Supplementary file 12 — Supplementary Table Descriptions [file 41380_2022_1484_MOESM12_ESM.docx]

**Supplementary Table 1.** Protein expression table containing a list of proteins detected and quantified in the mouse developmental time course experiment. The data are filtered for missing values.

**Supplementary Table 2.** Summary statistics table of the differential expression analysis of proteins detected in the mouse developmental time course experiment. The table contains test statistic, fold change and q-value information. Proteins, which are statistically significantly differentially expressed (i.e. q-value <= 0.05) in at least one time point, are marked.

**Supplementary Table 3.** Table of pathways enriched in the mouse developmental time course experiment. Pathway analysis was performed using the Perseus 1D enrichment analysis. Annotation terms are filtered such that a term is retaining if it is significantly up- or down-regulated in at least one time point after multiple hypothesis correction (Benj. Hoch. FDR <= 0.05).

**Supplementary Table 4.** Protein expression table containing a list of proteins detected and quantified in the rat brain regions experiment. The data are filtered for missing values.

**Supplementary Table 5.** Summary statistics table of the differential expression analysis of proteins detected in the rat brain regions experiment. The table contains test statistic, fold change and q-value information. Proteins, which are statistically significantly differentially expressed (i.e. q-value <= 0.05) in at least one brain region, are marked.

**Supplementary Table 6.** Protein expression table containing a list of proteins detected and quantified in the mouse Ube3a reinstatement experiment. The data are filtered for missing values.

**Supplementary Table 7.** Summary statistics table of the differential expression analysis of proteins detected in the mouse Ube3a expression reinstatement experiment. Proteins that are differentially expressed in at least one of 4 groups (Control, AS, Reinstatement at p21, and Ube3a Reinstatement at p56) are marked (q-value <= 0.05). Post-hoc analysis was performed with the Tukey’s Honest Significant Difference test and test-statistic and adjusted p-values are provided.

**Supplementary Table 8.** Table of pathways enriched in the mouse Ube3a expression reinstatement experiment. Pathway analysis was performed using the Perseus 1D enrichment analysis. All annotation terms are retained regardless of significance level. To subset to significantly enriched/depleted pathways, Benj. Hoch. FDR has to be set to <= 0.05.

**Supplementary Table 9.** Table of proteins from Fig. 4E and their subcellular localization in the human protein atlas.
